# Antibiotic duration for common bacterial infections—a systematic review

**DOI:** 10.1093/jacamr/dlae215

**Published:** 2025-01-29

**Authors:** Yin Mo, Wei Cong Tan, Ben S Cooper

**Affiliations:** Division of Infectious Diseases, University Medicine Cluster, National University Hospital, Singapore, Singapore; Department of Medicine, Yong Loo Lin School of Medicine, National University of Singapore, Singapore, Singapore; Saw Swee Hock School of Public Health, National University of Singapore, Singapore, Singapore; Centre for Tropical Medicine and Global Health, Nuffield Department of Medicine, University of Oxford, Oxford, UK; Mahidol-Oxford Tropical Medicine Research Unit, Faculty of Tropical Medicine, Mahidol University, Bangkok, Thailand; Division of Infectious Diseases, University Medicine Cluster, National University Hospital, Singapore, Singapore; Centre for Tropical Medicine and Global Health, Nuffield Department of Medicine, University of Oxford, Oxford, UK; Mahidol-Oxford Tropical Medicine Research Unit, Faculty of Tropical Medicine, Mahidol University, Bangkok, Thailand

## Abstract

**Background:**

Reducing antibiotic duration is a key stewardship intervention to mitigate antimicrobial resistance (AMR). We examined current evidence informing antibiotic duration for common bacterial infections to identify any gaps in terms of settings, patient populations and infectious conditions. Trial methodologies were assessed to identify areas for improvement.

**Methods:**

MEDLINE and Embase were searched up to July 2024 for randomized trials comparing antibiotic durations in hospital and community settings (PROSPERO 2021, CRD42021276209). A narrative synthesis of the results was performed with a review on the major guidelines published by IDSA, NICE, WHO and other international societies to assess the impact of these trials on practice guidance.

**Results:**

Out of 315 studies, 85% concluded equivalence or non-inferiority of shorter courses. Adult bacterial sinusitis, community-acquired pneumonia, female cystitis/pyelonephritis, uncomplicated cellulitis and intra-abdominal infection with adequate source control and perioperative prophylaxis had robust evidence supporting shorter durations. Few trials studied severe infections, such as bloodstream infections and ventilator-associated pneumonia. Twenty-three (7%) of the trials were conducted in intensive care settings and only 43 trials (14%) enrolled patients from low-to-middle- or low-income countries. Only 15% of studies were at low risk for bias.

**Conclusions:**

Reducing antibiotic duration likely remains an important strategy for antibiotic stewardship, and an area of active research. While shorter antibiotic courses may be suitable for many bacterial infections, more evidence is needed for severe infections and in low- and middle-income settings.

## Introduction

Human consumption of antibiotics is a major driver of antimicrobial resistance (AMR),^1^ and reducing unnecessary antibiotic use is a key intervention for reducing AMR. Increasing calls to view antibiotics as a non-renewable resource has given rise to systematic and evidence-based antibiotic stewardship programmes, which are strongly advocated by the WHO and international infectious disease authorities.^2^

Reducing antibiotic duration is one of the most commonly implemented antibiotic stewardship strategies reported in the literature,^3^ and the one that is deemed to be safest and most acceptable by practising clinicians.^4^ This includes shortening courses for established bacterial infections, preventing bacterial infections and rapid discontinuation of antibiotic prescriptions when bacterial infections have been excluded.

The conventional principle for antibiotic duration was to treat beyond clinical improvement in order to prevent both relapse of infection and development of antibiotic resistance. The message of always completing antibiotic courses to prevent the development of resistance has remained widespread until recent years, promoted by the WHO, international health authorities, national health campaigns and school curricula.^4,5^

However, this led to a parallel concern of excess antibiotic use. The spotlight on this and the emergence of widespread AMR in the late 1990s sparked numerous randomized controlled trials to shorten antibiotic duration. Aside from directly comparing durations, inflammatory markers such as serum C-reactive protein and procalcitonin, and individualized expert opinion such as infectious disease specialist consultations have also been used in trials to inform duration decisions.^6^

This systematic review focuses on all randomized controlled trials evaluating definitive antibiotic duration for common bacterial infections and perioperative prophylaxis in humans and aims to: (i) review current evidence for shorter-course antibiotic durations; (ii) identify gaps in evidence for antibiotic durations in terms of settings, patient populations and infectious conditions; and (iii) appraise trial methodologies and identify areas for improvement.

## Methods

### Data sources and searches

This systematic review was performed in accordance with the Preferred Reporting Items for Systematic Reviews and Meta-Analysis (PRISMA) guidelines (Supplementary material, available as Supplementary data at *JAC-AMR* Online). Randomized controlled trials published in the English language and indexed in MEDLINE or Embase were sought.^7^ Unpublished studies and preprints were excluded. This article includes surveillance through to 31 July 2024. This systematic review was registered on PROSPERO (reference: PROSPERO 2021, CRD42021276209).

### Study selection

A Boolean search strategy with search terms pertaining to antibiotic treatment, duration, bacterial infections, human health, and randomized controlled trials was adopted (Table S2). Additional relevant titles found in other systematic reviews were also included.

The specific inclusion criteria were: (i) patients from either hospital or community settings who received antibiotics for the prevention or treatment of bacterial infections; (ii) random assignment to trial arms with varying duration of antibiotic treatment (examples of the specific interventions used to guide antibiotic duration include, but are not limited to, the use of inflammatory markers, expert opinion or electronic prescription systems); and (iii) clinical outcomes (e.g. cure, relapse, mortality). The exclusion criteria were: (i) patients with non-infectious conditions, fungal or viral infections, or TB; (ii) intervention involving no antibiotic courses (e.g. comparison between antibiotic courses and a surgical procedure); and (iii) feasibility or pilot studies.

Y.M. and W.C.T. reviewed each abstract for potential full-text review independently. One investigator then reviewed each full-text article for inclusion, and a second verified the decision to exclude. Disagreements were resolved through consensus, with B.S.C. making the final decision.

### Data extraction and quality assessment

Full texts for all studies included in the systematic review were retrieved for data extraction. Data extracted from all included studies were: age group; type of bacterial infection; treatment durations; blinding; how duration was determined; and healthcare settings. To provide a more detailed assessment of current methodologies and aspects of trial design and conduct affecting risk of bias, additional data were extracted from trials conducted since 2006 (chosen arbitrarily). These additional data included: study hypothesis and sample size calculation; how the bacterial infection was identified; antibiotic choice; randomization process; follow-up period; non-adherence and how this was monitored; analysis methods; and types of outcomes reported. Randomized trials published from 2006 onwards, and which achieved the target enrolment number, were further assessed according to the key domains outlined in the revised tool for assessing risk of bias in randomized trials (RoB 2).^8^

Y.M. and W.C.T. reviewed the shortlisted articles and extracted the underlying data for the systematic review independently. Both authors verified the extracted data. Conflicts during the screening and data extraction processes were resolved through discussion, with B.S.C. making the final decision.

### Data synthesis and analysis

Quantitative synthesis was not possible owing to heterogeneity in types of bacterial infections, study design, patient populations, comparisons and analytic methods. Instead, we performed a narrative synthesis of the results.

In addition, we reviewed major guidelines published by IDSA, NICE, WHO and other national/international societies to assess the impact of the randomized trials on practice guidance.

## Results

### Overview of antibiotic duration randomized trials

**Figure 1.**
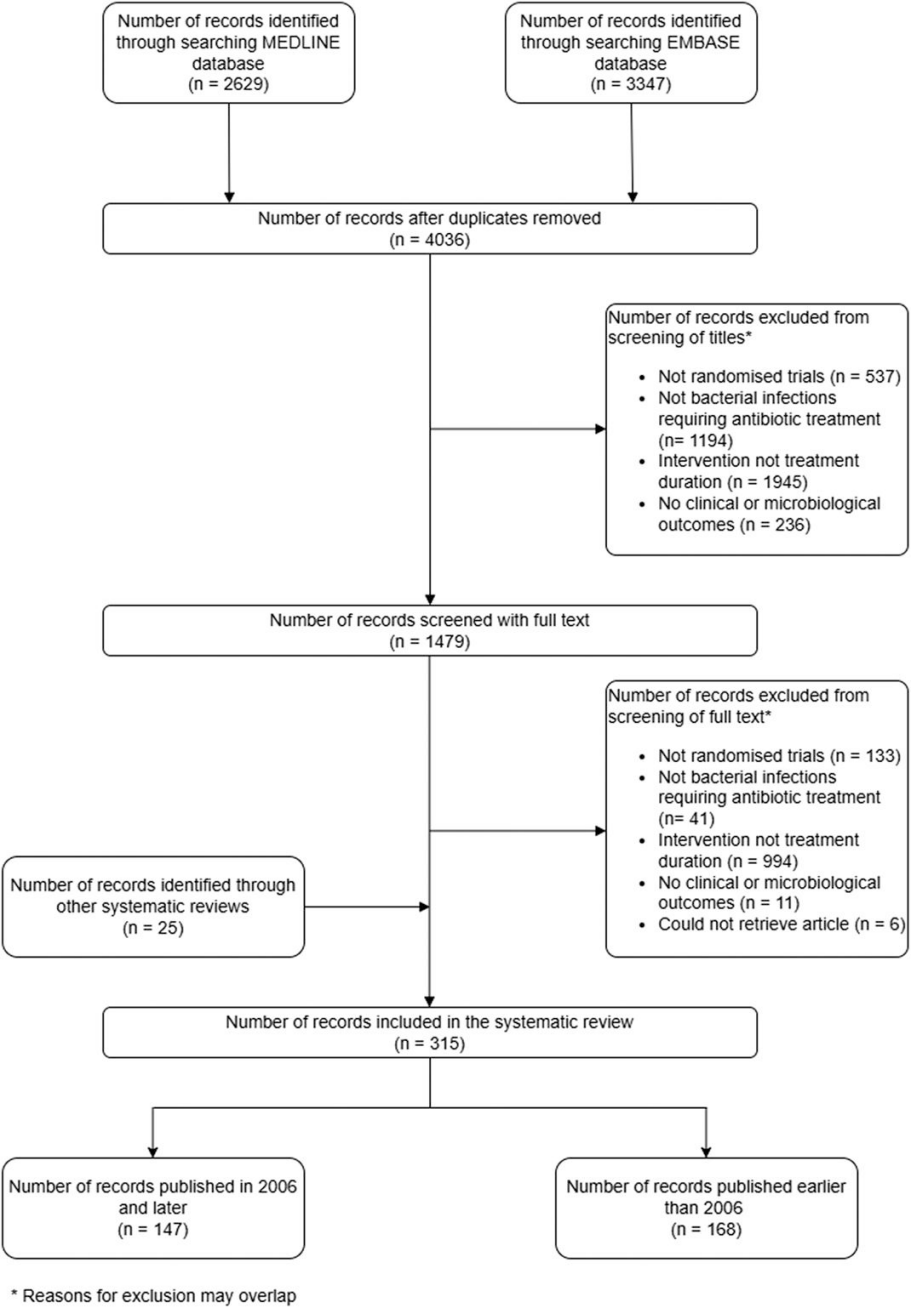
PRISMA flow diagram.

**Figure 2.**
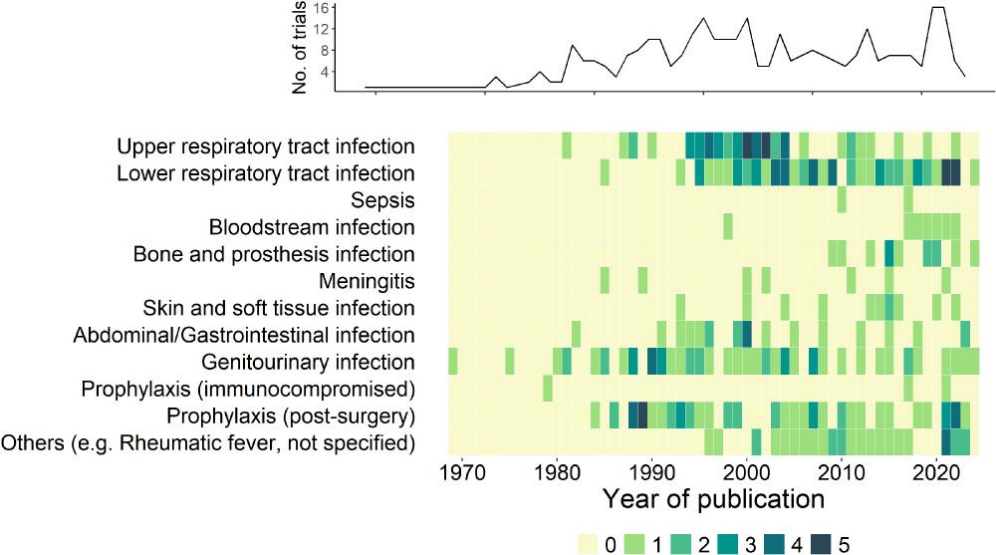
Types of bacterial infections studied by antibiotic duration randomized trials over time. The top panel shows the total number of published randomized trials per year from 1969 to 31 July 2024. The bottom panel shows the number of trials for each type of infection over time. The shading intensity of the boxes represents the number of trials per year.

The initial electronic database search produced 4036 unique records published from 1969 to 31 July 2024 (Figure 1). Of these, 315 fulfilled the inclusion criteria. The most frequently studied bacterial infections were upper (52/315; 17%) and lower (66/315; 21%) respiratory tract infections and genitourinary infections (52/315; 17%). Perioperative prophylaxis (52/315; 17%) was also extensively studied (Figure 2). Eighty-five percent (267/315) of the trials concluded that there was either no statistical difference, equivalence or non-inferiority between the short and long antibiotic courses when clinical outcomes were compared.

**Figure 3.**
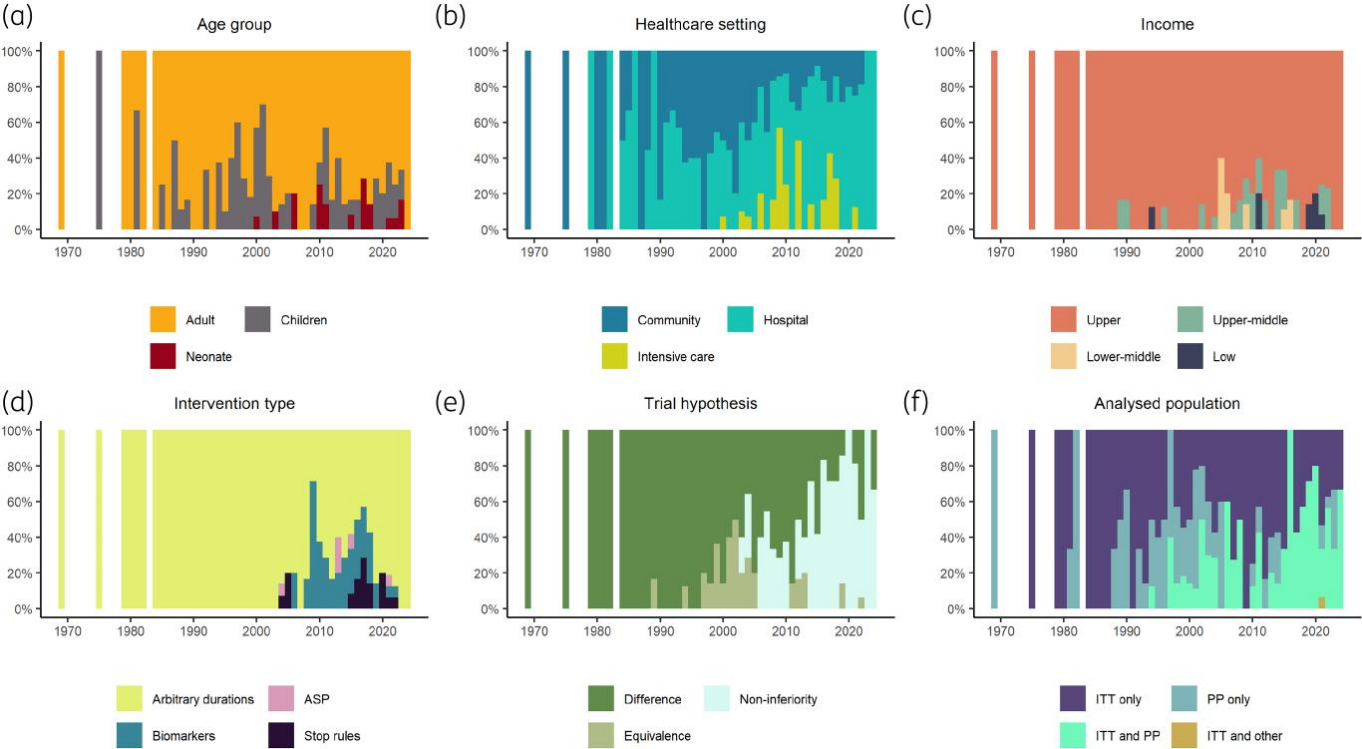
Characteristics of antibiotic duration trials over time. Each panel is labelled with a trial characteristic: (a) age group of the trial participants; (b) the healthcare setting that the participants were enrolled from; (c) the minimum income level of the country/countries where the participants were enrolled from; (d) the type of intervention studied in the trials; (e) the trial hypothesis design; and (f) the participant populations that the conclusions of the trials were based on. The proportion of the trials published each year with a certain characteristic (*y*-axis) is plotted against the year of publication (*x*-axis) to illustrate the changes in these trial characteristics over time. ASP, antibiotic stewardship programme.

Most antibiotic treatment duration trials prior to 2004 exclusively tested various arbitrary durations (Figure 3). Biomarkers such as procalcitonin and C-reactive protein were increasingly studied in the 2000s but declined from the mid-2010s. Another type of intervention is the use of stopping rules incorporating individual clinical response.

In terms of patient characteristics, the most frequently represented groups consisted of adults from either the hospital general ward or the community in upper- and upper-middle-income countries (Figure 3). Only 23 (7%) of the trials were conducted in intensive care settings, and 43 trials (14%) enrolled patients from low- and middle- or low-income countries.

### Evidence from antibiotic treatment duration randomized trials for specific bacterial infections

**Figure 4.**
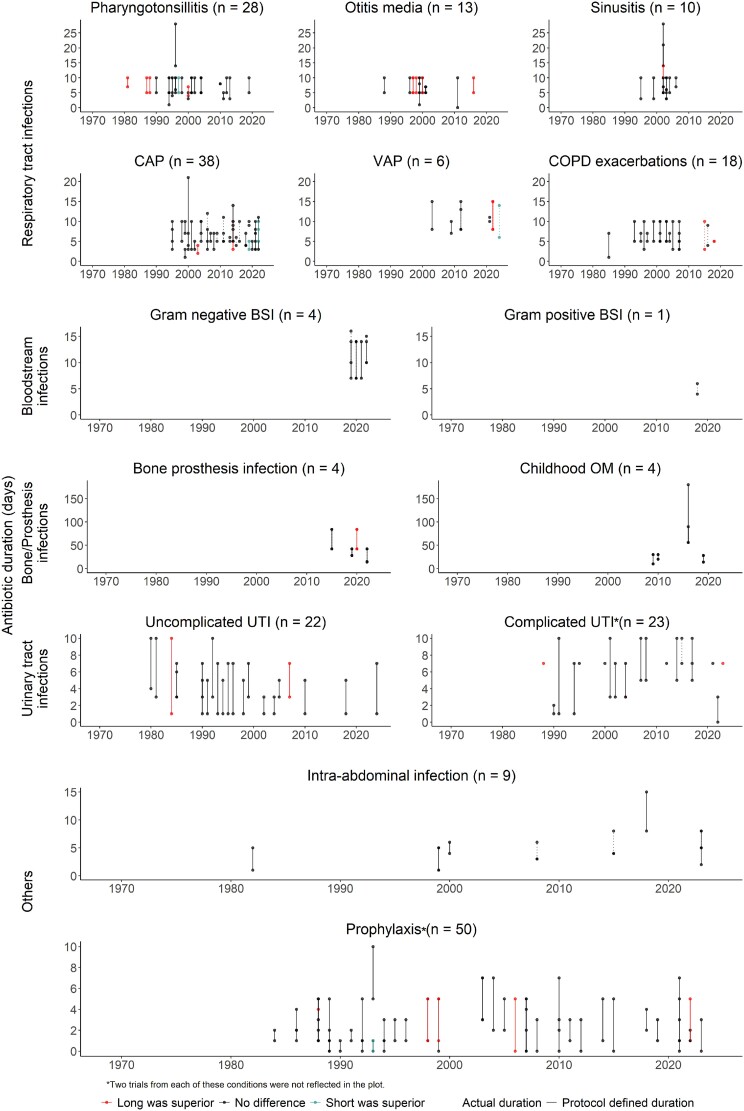
Antibiotic duration trials classified by bacterial infection syndromes. Each plot presents trial results for a type of bacterial infection. The number of trials included in each plot is shown in brackets. The vertical lines joining the points in each plot represent the durations compared in each trial. Line colours indicate whether trials concluded that long duration was superior to short duration (orange), not different or non-inferior (black) or inferior (teal) in terms of clinical outcomes. Solid vertical lines show the durations allocated to the study participants (plotted only when a trial defined arbitrary durations as the intervention). Dotted vertical lines show the actual mean duration observed during the trial (plotted only when reported). Studies represented by single points were trials that used procalcitonin but failed to reduce antibiotic durations. Complicated UTI encompassed simple cystitis in males, catheter-associated UTI and pyelonephritis. The single trial shown in the Gram-positive BSI panel studied staphylococcal bloodstream infection (BSI). Only trials that reported antibiotic durations were included in this figure. Four trials were excluded from this figure as the antibiotic durations in these trials were vastly different from the other trials within the same infection syndrome.^124–127^ CAP, community-acquired pneumonia; VAP, ventilator-associated pneumonia; UTI, urinary tract infection; OM, osteomyelitis.

Evidence for antibiotic treatment durations for common bacterial infections are discussed in detail below (Figure 4). Specific recommendations from major guidelines are included in Table 1.

**Table 1.** Summary of antibiotic duration recommendations for bacterial infections from major guidelines

Infection syndrome	Major guidelines^a^ Recommendation, year of publication, [strength of recommendation as stated in the guideline]				Comments
	US guideline	UK guideline	European guideline	WHO/international guidelines	
Respiratory tract					
Acute GAS pharyngotonsillitis	10 days penicillin or amoxicillin (first line) [Strong recommendation, high evidence], first generation cephalosporin, clindamycin or clarithromycin [strong recommendation, moderate evidence]; 5 days azithromycin (IDSA, 2012)	5–10 days phenoxymethylpenicillin (first line); 5 days clarithromycin or erythromycin (second line) in adults and children [Very low to low-quality evidence] (NICE, 2018)	Not available	5 days (low risk^b^) or 10 days (high risk^b^) amoxicillin or phenoxymethylpenicillin (first line); 5 days cefalexin or clarithromycin (second line) (AWaRe antibiotic book 2023)	5 days may be adequate for symptomatic cure; 10 days is associated with higher rates of GAS pharyngeal eradication
Otitis media in children	10 days amoxicillin or amoxicillin/clavulanate (first line) in <2 years or severe symptoms; 7 days first-line antibiotics in 2–5 years with mild or moderate symptoms; 5–7 days first-line antibiotics in >6 years with mild to moderate symptoms (AAP/AAFP, 2013)	5–7 days amoxicillin (first line), clarithromycin or amoxicillin/clavulanate in 1 month to 17 years, with 7 days reserved for those with severe or recurrent infection [Very low- to low-quality evidence] (NICE, 2018)	5–10 days [Weak evidence] (Systematic review of guidelines across 17 countries in the EU, 2020)	5 days amoxicillin (first line) or amoxicillin/clavulanate (AWaRe antibiotic book, 2023)	
Acute bacterial sinusitis	In adults, 5–7 days amoxicillin/clavulanate (first line) or doxycycline In children, 10–14 days amoxicillin/clavulanate (first line) [Weak recommendation, low–moderate evidence] (IDSA, 2012)	In adults and children, 5 days phenoxymethylpenicillin or amoxicillin/clavulanate (first line), doxycycline or erythromycin [Moderate to high evidence] (NICE, 2017)	No specific recommendations on antibiotic duration [Weak evidence] (European position paper, 2020)	5 days amoxicillin (first line), or amoxicillin/clavulanate (AWaRe antibiotic book, 2023)	Longer duration recommended in some guidelines for paediatric patients because shorter course antibiotic has not been studied in this population with randomized trials
Community-acquired pneumonia	In adults, 5 days amoxicillin, doxycycline or macrolide (if local pneumococcal resistance is <25%, no comorbidities or risk factors for MRSA or *P. aeruginosa*) or more until clinical stability; 7 days for MRSA or *P. aeruginosa* pneumonia [Strong recommendation, moderate evidence] In children, no specific recommendation on antibiotic duration (IDSA, 2019)	In adults and children, 5 days amoxicillin (first line), doxycycline, clarithromycin or erythromycin for mild infections; combine amoxicillin or amoxicillin/clavulanate with doxycycline, clarithromycin or erythromycin for moderate to severe infections (avoid doxycycline in children <12 years) (NICE, 2019)	8 days or less in responding patient [1 RCT or more, consistent evidence/clear outcome]; (ERS/ESCMID, 2011) Addition of 3–5 days of macrolides to β-lactams in hospitalized patients with severe infections [Conditional recommendation, very low evidence] (ERS/ESICM/ESCMID/ALAT, 2023)	In adults, 5 days amoxicillin or phenoxymethylpenicillin (first line), amoxicillin/clavulanate or doxycycline until clinical stability for mild–moderate infections; cefotaxime or ceftriaxone (first line), or amoxicillin/clavulanate with or without clarithromycin for severe infections In children from areas of low HIV prevalence and no chest indrawing, 3 days amoxicillin for mild to moderate infections; longer treatment until clinical stability with IV amoxicillin, ampicillin, or benzylpenicillin with gentamicin for severe infections (AWaRe antibiotic book, 2023)	
Ventilator-associated pneumonia	7 days [Strong recommendation, moderate evidence] (IDSA, 2016)	Not available	7–8 days in patients without immunodeficiency, cystic fibrosis, empyema, lung abscess, cavitation or necrotizing pneumonia and with a good clinical response to therapy [Weak recommendation, moderate evidence] (ERS/ESICM/ESCMID/ALAT, 2017)	Not available	
Bacterial exacerbation of COPD	Not available	5 days amoxicillin, doxycycline or clarithromycin (first line), amoxicillin/clavulanate, trimethoprim/sulfamethoxazole or levofloxacin [Low to moderate evidence] (NICE, 2018)	No specific recommendation on antibiotic duration (ERS/ATS, 2017)	5 days amoxicillin (first line), cefalexin or doxycycline in mild–moderate infections, amoxicillin/clavulanate in severe infections (AWaRe antibiotic book, 2023)	
Genitourinary tract					
Simple cystitis in women	5 days nitrofurantoin; 3 days trimethoprim/sulfamethoxazole (first line); 3 days trimethoprim or fluoroquinolones; single-dose fosfomycin; 3–7 days pivmecillinam or β-lactam agents [Moderate to good evidence] (IDSA, 2011)	3 days nitrofurantoin or trimethoprim (first line); 3 days pivmecillinam; single-dose fosfomycin in non-pregnant women >15 years 7 days nitrofurantoin (first line); amoxicillin or cefalexin for pregnant women >11 years [Very low to moderate evidence] (NICE, 2018)	Single-dose fosfomycin; 3–5 days pivmecillinam; 5 days nitrofurantoin [Strong recommendation, 1 RCT or more] (EAU, 2023)	3–5 days amoxicillin/clavulanate; 5 days nitrofurantoin (preferred); 3 days trimethoprim/ sulfamethoxazole or trimethoprim; consider longer treatment in pregnant women, i.e. 5 days (AWaRe antibiotic book, 2023)	
Complicated urinary tract infections (in males, catheter-associated, upper urinary tract involvement)	Catheter-associated: Up to 14 days depending on symptom resolution [Good evidence, expert opinion]; 5 days levofloxacin for mild infections [Moderate evidence, expert opinion]; 3 days for women <65 years without upper tract involvement after indwelling catheter removal [Moderate evidence, 1 well-designed observational study or more] Uncomplicated pyelonephritis: 7 days ciprofloxacin [Good evidence, 1 RCT or more]; 5 days levofloxacin [Moderate evidence, 1 well-designed observational study or more]; 14 days trimethoprim/ sulfamethoxazole [Good evidence, 1 RCT or more] (IDSA, 2010)	Catheter-associated: 7 days nitrofurantoin, trimethoprim, amoxicillin (first line), or pivmecillinam in non-pregnant women and men >16 years if upper tract involvement; 7–10 days cefalexin or amoxicillin/clavulanate; 14 days trimethoprim; or 7 days ciprofloxacin (first line) if upper tract involvement Males: 7 days trimethoprim or nitrofurantoin (first line) for >16 years [Very low to moderate evidence] Acute pyelonephritis in men >16 years: 7–10 days cefalexin or amoxicillin/clavulanate (first line); 14 days trimethoprim; 7 days ciprofloxacin (NICE, 2018)	Catheter-associated: Up to 14 days depending on symptom resolution, indwelling catheter removal; 5 days levofloxacin for mild infections [Strong recommendation, 1 RCT or more] Uncomplicated pyelonephritis: 7 days ciprofloxacin; 5 days levofloxacin; 14 days trimethoprim/ sulfamethoxazole; 10 days cefpodoxime or ceftibuten Males with prostatitis: 14 days (EAU, 2023)	Uncomplicated pyelonephritis: 7 days ciprofloxacin in mild infections; cefotaxime, ceftriaxone and/or amikacin and/or gentamicin in severe infections (AWaRe antibiotic book, 2023)	
Bone and joint infections					
Adult osteomyelitis, septic arthritis and prosthetic joint infection	Osteomyelitis: MRSA: ≥ 8 weeks [Good evidence, 1 well-designed observational study or more] Septic arthritis: MRSA: 3–4 weeks with debridement or drainage [Good evidence, expert opinion] (IDSA, 2011) Prosthetic joint infection: Following debridement and prosthesis retention or 1-stage exchange: *Staphylococcus*: 2–6 weeks IV antibiotic with rifampicin (extend to 4–6 weeks if rifampicin cannot be used), followed by oral antibiotic with rifampicin for a total of 3 months for hip; 6 months for knee [Good evidence, 1 RCT or more] Other organism(s): 4–6 weeks of IV or highly bioavailable oral antibiotics [Moderate evidence, 1 well-designed observational study or more] Indefinite chronic oral antimicrobial suppression for retained prosthesis [Moderate evidence, expert opinion] Following resection arthroplasty with or without planned staged reimplantation: 4–6 weeks post procedure [Good evidence, 1 well-designed observational study or more] Following amputation and complete removal of infected tissue: 1–2 days post procedure [Poor evidence, expert opinion] (IDSA, 2014)	Osteomyelitis: *Staphylococcus*: 6 weeks flucloxacillin or clindamycin, or vancomycin or teicoplanin (if MRSA suspected) with fusidic acid or rifampicin for initial 2 weeks Septic arthritis: *Staphylococcus*: 4–6 weeks *Neisseria gonorrhoeae* or Gram-negative: 4–6 weeks cefotaxime or ceftriaxone (NICE, 2020)	Septic arthritis: Native joint: 1 to 2 weeks of IV antibiotics followed by 2–4 weeks oral antibiotics [Very low-quality, weak recommendation] After reconstruction of anterior cruciate ligament: 1–2 weeks of IV antibiotics followed by 4–5 weeks of oral antibiotics [Very low-quality, weak recommendation] (EBJIS, 2023) Prosthetic joint infection: 4–6 weeks (Italy, 2006)	Osteomyelitis: 4–6 weeks cloxacillin (first line), amoxicillin/clavulanate, cefazolin, cefotaxime, ceftriaxone or clindamycin Septic arthritis: 4–6 weeks cloxacillin (first line), amoxicillin/clavulanate, cefazolin, cefotaxime, ceftriaxone or clindamycin *N. gonorrhoeae:* 2 weeks (AWaRe antibiotic book, 2023)	
Childhood osteomyelitis/septic arthritis	MRSA osteomyelitis: MRSA 4–6 weeks [Good evidence, 1 well-designed observational study or more] MRSA septic arthritis: 3–4 weeks [Good evidence, 1 well-designed observational study or more] (IDSA, 2011)	Not available	Uncomplicated native joint septic arthritis: 2–4 days IV antibiotics with total antibiotic duration of 2–3 weeks (EBJIS, 2023)	Osteomyelitis (with uncomplicated infections) and septic arthritis: 3 weeks cloxacillin (first line), amoxicillin/clavulanate, cefazolin, cefotaxime, ceftriaxone or clindamycin	
Others					
Bloodstream infections	Varied durations depend on bacterial pathogen, source control and secondary seeding: 4–6 weeks for persistent bacteraemia after catheter removal, complications of infective endocarditis or suppurative thrombophlebitis, complicated staphylococcal bacteraemia, paediatric patients with osteomyelitis [Good evidence] 2 weeks for uncomplicated staphylococcal bacteraemia (exclusion of endocarditis, no implanted prostheses, clearance of follow-up blood cultures 2–4 days after the initial set, defervescence within 72 h effective therapy, and no metastatic sites of infection) [Good evidence (IDSA guideline on intravascular catheter-related infection, 2009; guideline on MRSA infections, 2011)	In neonates, 7 days in early- (first 72 h after birth) and late-onset (after 1st week of birth to 3 months) sepsis if without meningitis, >7 days if not yet recovered or due to the identified pathogen; <7 days in prompt recovery and pathogen identified is common commensal or no pathogen identified (NICE guideline on neonatal infection, 2021)	Varied durations depend on the organism identified, the presence of complications and whether the intravascular catheter has been removed: 4–6 weeks if there is: prolonged or persistent bacteraemia after intravascular catheter removal (i.e. >72 h after removal), endocarditis or suppurative thrombophlebitis, metastatic focus of infection, osteomyelitis in paediatric patients (Ireland, 2014)	Not available	
Non-tuberculous meningitis	*Neisseria meningitidis*: 7 days third-generation cephalosporin (first line), penicillin G, ampicillin, chloramphenicol, fluoroquinolone, aztreonam *Haemophilus influenzae:* 7 days third-generation cephalosporin (first line), chloramphenicol, cefepime, meropenem, fluoroquinolone *Streptococcus pneumoniae:* 10–14 days vancomycin plus a third-generation cephalosporin (first line), meropenem, fluoroquinolone *Streptococcus agalactiae*: 14–21 days ampicillin or penicillin G (first line), third-generation cephalosporin Aerobic Gram-negative bacilli: 21 days third-generation cephalosporin (first line), cefepime, meropenem, aztreonam, fluoroquinolone, trimethoprim/sulfamethoxazole *Listeria monocytogenes*: ≥21 days ampicillin or penicillin G (first line), trimethoprim/sulfamethoxazole, meropenem [Good evidence, expert opinion] (IDSA, 2004)	>3 months: *H. influenzae* type B: 10 days ceftriaxone *S. pneumoniae:* 14 days ceftriaxone Unconfirmed bacterial meningitis: >10 days ceftriaxone <3 months: *Group B Streptococcus pneumonia:* >14 days cefotaxime or benzylpenicillin with gentamicin for first 5 days Gram-negative bacilli: >21 days cefotaxime *L. monocytogenes*: 21 days IV amoxicillin or ampicillin and gentamicin for at least the first 7 days Unconfirmed bacterial meningitis: >14 days cefotaxime plus ampicillin or amoxicillin (NICE, 2010 and 2021)	*N. meningitidis*: 7 days *S. pneumoniae*: 10–14 days *H. influenzae*: 7–10 days *S. aureus*: >14 days [Strong recommendation] *L. monocytogenes*: >21 days (ESCMID, 2016)	*N. meningitidis*: 5–7 days *S. pneumoniae*: 10–14 days *Listeria* meningitis: 21 days Unknown pathogen: 10 days cefotaxime or ceftriaxone (first line), amoxicillin, ampicillin, benzylpenicillin or chloramphenicol in adults and older children; 3 weeks in neonates <1 month, ampicillin with gentamicin, cefotaxime or ceftriaxone with gentamicin (first line), or meropenem (AWaRe antibiotic book, 2023)	
Cellulitis and skin abscesses	Cellulitis: 5 days [Strong recommendation, high evidence] Recurrent cellulitis (>3 episodes a year): 4–52 weeks oral penicillin or erythromycin; or every 2–4 weeks intramuscular benzathine penicillin [Strong recommendation, moderate evidence] Recurrent abscess: 5–10 days [Weak recommendation, low evidence] (IDSA, 2014)	Cellulitis: 5–7 days flucloxacillin (first line), clarithromycin, erythromycin, doxycycline; 7 days amoxicillin/clavulanate (first line) or clarithromycin and metronidazole if infection near eyes or nose (avoid doxycycline in children) [Limited evidence] (NICE, 2019)	Not available	Cellulitis: 5 days amoxicillin/clavulanate, cefalexin or cloxacillin in adults and children (AWaRe antibiotic book, 2023)	
Intra-abdominal infections	4–7 days with adequate source control [Moderate evidence] (IDSA, 2010)	Not available	2–3 days in localized community-acquired intra-abdominal infections with adequate source control, adapted to the degree of contamination observed intraoperatively [Strong agreement, weak recommendation]; 5–7 days in generalized community-acquired intra-abdominal infections [Strong agreement, weak recommendation] (SRLF, 2015)	<4 days with adequate source control, <7 days in patients with secondary bacteraemia who have undergone adequate source control and are no longer bacteraemic [Weak recommendation, moderate-quality evidence] 4 days in complicated appendicitis, complicated cholecystitis, diffuse peritonitis, small bowel perforation, gastroduodenal ulcer perforation, if source control is adequate in immunocompetent patients without sepsis 3–5 days in acute cholangitis with biliary drainage, or diverticular abscesses (with percutaneous drainage in abscesses >4–5 cm) 5–7 days in uncomplicated diverticulitis in immunocompromised patients or with sepsis (no antibiotics needed for uncomplicated diverticulitis otherwise), or in established intra-abdominal infections where definitive source control procedure is not performed [Weak recommendation, weak-quality evidence] <24 h in patients with traumatic bowel perforations, acute or gangrenous appendicitis/cholecystitis in absence of perforation [Strong recommendation, high-quality evidence], gastroduodenal perforations, ischaemic and non-perforated bowel [Strong recommendation, weak-quality evidence] (SIS, 2017; WSES/GAIS/SIS-E/WSIS/AAST, 2021)	
Perioperative prophylaxis	Generally single dose or continuation <24 h after end of surgery, with variations across types of surgical procedures, e.g. no need for prophylaxis in clean orthopaedic procedures not involving implantation of foreign materials [Evidence from well-conducted cohort studies or randomized trials] (IDSA, 2013)	Single dose on starting anaesthesia or earlier for operations in which a tourniquet is used, repeat dose when the operation is longer than the half-life of the antibiotic given (NICE, 2020)	Single dose if surgery lasts <4 h and no significant blood loss, total duration should not exceed 24 h after the end of surgery [Low to moderate evidence] (ECDC, 2013)	Single-dose cefazolin (first line) within 120 min of surgery, add metronidazole if contamination expected (AWaRe antibiotic book, 2023)	

Note that these recommendations are meant for when antibiotics are indicated or the decision for prescribing antibiotics has been made. Antibiotics may not be indicated in these infections. When antibiotic names are included without specific bacterial pathogens, they refer to empirical choices stated in the respective guidelines without the need for microbiological cultures. When antibiotic names are not included, they were not mentioned in the guidelines or culture directed antibiotics are required.

WHO AWaRe antibiotic book, WHO Access, Watch, Reserve antibiotic book; AAP, American Academy of Pediatrics; AAFP, American Academy of Family Physicians; ERS, European Respiratory Society; ESICM, European Society of Intensive Care Medicine, ESCMID, European Society of Clinical Microbiology and Infectious Diseases; ALAT, Latin American Thoracic Association; ATS, American Thoracic Society; EAU, European Association of Urology; RCT, randomized controlled trial; EBJIS, European Bone and Joint Infection Society; SRLF, *Société de réanimation de langue française*; SIS-E, Surgical Infection Society-Europe; WSES, World Society of Emergency Surgery; GAIS, Global Alliance for Infections in Surgery; WSIS, World Surgical Infection Society; AAST, American Association for the Surgery of Trauma.

^a^Full reference list can be found in the Supplementary material (references).

^b^Risk of GAS pharyngitis can be estimated by the Centor score^+^ local prevalence or previous history of rheumatic fever. Centor score is the sum of: presence of fever, absence of cough, tonsillar exudates or swelling, and tender anterior cervical lymphadenopathy, where each criterion is given 1 point (<2 points = low risk, 2–3 moderate risk, >3 high risk of GAS pharyngitis).

#### Respiratory tract infections

##### Acute group A Streptococcus (GAS) pharyngotonsillitis

Twenty-eight trials studied antibiotic duration for GAS pharyngotonsillitis. Important outcomes in these trials included both symptom resolution and microbiological eradication to prevent immunological sequelae such as rheumatic fever. A short course of 5–7 days phenoxymethylpenicillin was found to have a lower microbiological cure rate compared with 10 days of treatment [number needed to treat 13 (range 8–37)] in a single systematic review and meta-analysis published in 2008.^9^ Hence, IDSA recommended 10 days of penicillin or amoxicillin,^10^ while WHO and NICE recommended 5–10 days depending on the risk of GAS as the cause for pharyngotonsillitis.^11,12^

In contrast to most studies involving phenoxymethylpenicillin, which used 10 day courses, shorter courses of 5 days of macrolides (e.g. azithromycin, erythromycin) and late-generation cephalosporins (e.g. cefpodoxime, cefdinir) were studied.^13,14^ However, shorter-course cephalosporin studies lacked strict entry criteria, did not assess adherence to antibiotics, and did not differentiate the serotypes or genotypes between infection episodes that failed treatment versus those that were deemed to be newly acquired.^10^ In addition, macrolides and late-generation cephalosporins are broader in antimicrobial spectrum than penicillins and more costly. Therefore, 5 days of macrolides is recommended as second-line treatments, and late-generation cephalosporins were not recommended across major guidelines.^10–12^

There remain important evidence gaps, especially for evaluation of long-term clinical complications from reduced antibiotic treatment duration. No trial evaluated the effect of an alternative antibiotic to penicillin for preventing acute rheumatic fever. In addition, few trials were performed in low- and middle-income settings, where the prevalence of rheumatic fever is high.^15^

##### Otitis media in children

Thirteen trials studied antibiotic duration in otitis media amongst children. The conventional antibiotic treatment duration adopted in the trials was 10 days, which was compared with shorter durations of 5–7 days. These randomized trials showed varied results.^16–19^ Generally, in older children more than 2–5 years old, whose host immunity tends to be mature and the Eustachian tube spontaneously drains, shorter courses or no antibiotics might be needed.^20–22^ In a landmark placebo-controlled trial conducted in the USA, children younger than 2 years old were randomized between 10 and 5 days of amoxicillin/clavulanate.^16^ Children in the shorter-course arm had more clinical failure (34% versus 16%, difference 17%, 95% CI 9%–25%). In addition, prolonged antibiotics might be indicated for those with recurrent or severe infections.

The trials were heterogeneous in terms of diagnostic criteria, antibiotic choices (which may have different bioavailability) and definitions of symptom resolution.^23^ High rates of spontaneous resolution in many of these trials might have suggested that antibiotics might not have been required. Evidence gaps exist for older children older than 2 years, and in low- and middle-income countries, where prevalence of antibiotic resistance is higher.

##### Acute bacterial sinusitis

Ten trials studied antibiotic duration in acute bacterial sinusitis, performed exclusively in adults. A meta-analysis showed that there was no difference in cure or improvement between shorter-course (3–7 days) and longer-course (6–10 days) antibiotics in adults, and is associated with fewer antibiotic side effects.^24^ In children, IDSA recommended 10–14 days of antibiotics based on expert opinion as there was no randomized trial evidence,^25,26^ while NICE and WHO recommended 5 days only in those who are systematically unwell, with symptoms and signs of more serious infection or at high risk of complications.^12,27^

The trials used heterogeneous interventions, which included various adjunctive therapies for symptom relief. Enrolled patients were heterogeneous, due to varying symptom duration prior to antibiotic treatments, non-specific diagnostic criteria and lack of microbiological testing. These may have led to patients with viral sinusitis being enrolled into the trials.

##### Community-acquired pneumonia

There were 38 randomized trials that studied antibiotic duration in community-acquired pneumonia. Shorter-course antibiotics (5 days) were consistently shown to be non-inferior to longer durations, especially in mild to moderate infections in outpatient settings in children and adults.^28–32^ This result included all empirical antibiotic options for community-acquired pneumonia, i.e. macrolides, quinolones and β-lactams.^33^ All major guidelines recommended to prolong antibiotics in patients with delayed clinical response and severe disease, e.g. pulmonary or extrapulmonary complications, inadequate procalcitonin response or antibiotic-resistant organisms.^12,32,34–36^ However, no randomized trials were performed that defined antibiotic treatment duration for patients who might require longer-course antibiotics.

A wide variety of antibiotics with different bioavailability were compared within and between these trials. None of the trials compared the same antibiotic at the same daily dosage in the outpatient setting.^37^ Though there were studies that supported 3–5 day antibiotic courses for treatment in children with mild community-acquired pneumonia,^38–40^ lack of chest imaging and microbiological testing might have allowed patients with viral pneumonia or upper respiratory tract infections to be enrolled. There were no randomized controlled trials for radiographically confirmed pneumonia in children.^30,41,42^

##### Ventilator-associated pneumonia

There were six trials that studied antibiotic duration for ventilator-associated pneumonia, which all showed non-inferiority of shorter-course antibiotics (7–8 days) against 14 days.^43–48^ Hence, major guidelines recommended 7–8 days for patients with no pulmonary or extrapulmonary complications.^49,50^ While a higher rate of recurrence with shorter-course antibiotics was observed in patients with pneumonia caused by non-fermenting Gram-negative rods, such as *Pseudomonas aeruginosa*, a meta-analysis showed no increased risk of 60 day mortality or recurrence in this group and those associated with carbapenem resistance.^47,48,51,52^ Antibiotic duration could be further reduced to individual response to culture-directed antibiotics, as demonstrated by the REGARD-VAP (individualized, short-course antibiotic treatment versus usual long-course treatment for ventilator-associated pneumonia), specifically stable haemodynamics and resolution of fever.^52^

There were no specific diagnostic criteria used to define ventilator-associated pneumonia, which might have led to patients with no pneumonia being enrolled into the trials. Procalcitonin has been used in randomized trials to define antibiotic durations but it is yet to be routinely recommended by major guidelines.^49,50^ A meta-analysis of randomized trials performed in 2018 showed that using procalcitonin to stop antibiotics in ventilator-associated pneumonia reduced treatment duration from 13 to 11 days.^53^

##### Bacterial exacerbation of COPD

There were 18 trials that studied antibiotic duration in infective exacerbations of COPD. These trials found that shorter-course antibiotics (less than 6 days) were comparable to longer courses (7 days or more of the same antibiotic) in terms of symptom resolution, and were associated with fewer antibiotic side effects.^54^ Shorter-course antibiotics were comparable to longer courses when evaluated using outcomes at 1, 2 and 3 weeks, and in both inpatient and outpatient settings.^54^

These trials adopted highly heterogeneous diagnostic criteria for COPD, e.g. smoking exposure, airflow obstruction and exacerbation episodes were not all accompanied by microbiological confirmation. This may have led to patients with bronchiectasis and chronic asthma being enrolled into the trials.

#### Genitourinary tract infections

##### Simple cystitis in women

There were 22 trials that studied antibiotic duration in female simple cystitis. Shorter-course antibiotics, e.g. 5 days of nitrofurantoin or 3 days of trimethoprim/sulfamethoxazole, were supported by numerous randomized trials across non-pregnant women of different age groups. In pregnant women, the consensus across major guidelines was for 5–7 days,^12,55–57^ in view of potentially severe complications in this group, e.g. upper tract involvement and negative consequences for the neonate, and higher chances of microbiological eradication with a longer antibiotic course.^56^ None of the antibiotic-duration randomized trials for urinary tract infection enrolled pregnant women.

Increasing prevalence of AMR (e.g. trimethoprim/sulfamethoxazole resistance, ESBL production) among common bacteria that cause urinary tract infections were important considerations in treatment guideline recommendations.^12,55^

##### Complicated urinary tract infections (in males, catheter-associated, upper urinary tract involvement)

There were 25 trials that addressed antibiotic duration in complicated urinary tract infections. Urinary tract infections in males are considered to be at higher risk of complications than in females due to the potential development of outlet obstructions, upper tract involvement and progression to bloodstream infections.^58^ One randomized trial concluded that 7 days of ciprofloxacin or trimethoprim/sulfamethoxazole was non-inferior to 14 days (symptom resolution 93% in the 7 day group versus 90% in the 14 day group, difference 3%, lower limit of one-sided 97.5% CI 5%).^59^ However, enrolled patients were afebrile and did not have microbiological confirmation from urine samples.^59^ Another recent study compared 7 days or continued ofloxacin for 14 days in men with febrile urinary tract infections. This double-blinded, placebo-controlled study failed to demonstrate non-inferiority of the shorter-course treatment [56% in the 7 day group versus 78% in the 14 day group met treatment success, risk difference −21.9 (95% CI −33.3 to −10.1)].^60^ This trial was underpowered as treatment failure was lower than expected.

As for catheter-associated urinary tract infections, one randomized trial found 5 days of levofloxacin was non-inferior to 10 days of ciprofloxacin in complicated urinary tract infections (72% of the total patients enrolled, number of catheter-associated urinary tract infections not reported) and acute pyelonephritis (29% of the total patients enrolled) with a non-inferiority margin of 15% (91% in the 5 day levofloxacin group versus 87% in the 10 day ciprofloxacin group, 95% CI −10% to 1%).^61^ In the second trial, which studied exclusively catheter-associated urinary tract infections in patients with spinal cord injury, 5 day antibiotic with catheter exchange was compared with 10 day antibiotic with catheter retention.^62^ All patients achieved clinical cure at the end of therapy, but more patients in the shorter-course group had recurrent urinary tract infections than in the long-course group (32% versus 11%, *P* = 0.043). Catheter change prior to antibiotic administration, which may reduce the need for prolonged antibiotics, was studied in a single randomized trial involving 54 older adults in a long-stay care facility.^63^ This study found a significant difference in cure or improvement, favouring catheter change at 72 h (93% versus 41%), 28 days (89% versus 59%), but not at 7 days.^63^ Given this limited, low-quality evidence for antibiotic treatment of catheter-associated urinary tract infections, the guidelines recommended 7–14 days depending on clinical response and antibiotic choice, based on expert opinion.^64,65^

For females with pyelonephritis, 7 days of ciprofloxacin was found to be superior to 14 days of trimethoprim/sulfamethoxazole in terms of bacteriological and clinical cure rates.^66^ As there were no randomized trials for shorter-course β-lactams or trimethoprim/sulfamethoxazole, a course of 7–14 days was recommended in the guidelines.^12,56,64,65^

No trials evaluated antibiotic duration in microbiologically confirmed male urinary tract infections. The role of catheter exchange or removal remains unclear in catheter-associated urinary tract infections. Acute complicated urinary tract infections included in the trials to date were highly heterogeneous due to the presence of nephrolithiasis, tumours or implants, various sites of infection within the urinary tract and infection severity.

#### Bone and joint infections

##### Adult osteomyelitis/septic arthritis and prosthetic joint infection

Eleven trials evaluated antibiotic duration for osteomyelitis, septic arthritis or prosthetic joint infection in adults. Four studies focused on prosthetic joint infections, four on diabetic foot osteomyelitis, two on native joint septic arthritis and one on vertebral osteomyelitis. Interventions varied in terms of surgical debridement and one- or two-staged exchange arthroplasty. In the largest trial, which included patients with microbiologically confirmed prosthetic joint infection, 6 weeks was not non-inferior to 12 weeks of antibiotic therapy (persistent infection 18% in the 6 week group versus 9% in the 12 week group, risk difference 9%, 95% CI 2%–16%) within 2 years follow-up. Outcomes in the shorter-course group were worse, and mainly accounted for by those patients who had implant retention (31% versus 15%).^67^ Due to limited, low-quality evidence, there was lack of consensus in the guidelines.^12,68–74^ and expert opinion was to prolong antibiotic treatment for up to 12 weeks if implants were retained.^75^ In addition, the general consensus for retained infected implants was life-long antibiotic suppression therapy.^76^

In diabetic foot osteomyelitis, 3 weeks of antibiotic therapy post-debridement was shown to be non-inferior to 6 weeks in terms of clinical remission with a 25% margin (84% in the 3 week group versus 73% in the 6 week group) at 2 month follow-up.^77^ Two trials found antibiotics could be stopped early in those with mild infection (no need for surgical debridement) and without severe peripheral arterial disease.^78,79^ In one, exact antibiotic durations administered were not reported.^78^ In the second, 6 weeks was comparable to 12 weeks of antibiotics in terms of remission (12/20 versus 14/20, respectively, *P* = 0.50). For vertebral osteomyelitis, 6 weeks was non-inferior to 12 weeks of antibiotics for clinical cure (160/176; 91% in the 6 week group versus 159/175; 91% in the 12 week group, difference 0.05%, 95% CI −6.2% to 6.3%).^80^

A single randomized trial studied antibiotic duration in native joint septic arthritis post-surgical drainage, where the majority (99/154; 64%) involved hand and wrist joints and the commonest organism was MSSA and *Streptococcus* spp.^81^ Infection recurred in one patient in the 2 week arm and two in the 4 week arm (97% cure rate) within a median 2 years of follow-up.

Bone and joint infections are highly heterogeneous in terms of joints involved, infection severity and complications, time from symptom onset to surgical debridement, and bacterial pathogens. This made defining inclusion and exclusion criteria challenging. Interventions applied in these trials were also varied, e.g. single versus two-stage arthroplasty, removal versus retention of implants, requirement for surgical debridement, and different antibiotic regimens with variable bioavailability.^82^

##### Childhood osteomyelitis/septic arthritis

Two trials studied antibiotic duration for osteomyelitis or septic arthritis in children. Both were conducted in Finland. The primary outcomes were clinical cure and recurrence. Patients with acute osteomyelitis caused primarily by MSSA were given clindamycin or a first-generation cephalosporin for 10 days versus 30 days (first trial) or 20 days versus 30 days (second trial) after an initial IV treatment of 2–4 days in both groups.^83^ Antibiotics were discontinued with clinical symptom resolution and serum C-reactive protein normalization. Both studies showed comparable outcomes in shorter- versus longer-course groups.

Due to limited randomized trial evidence, there is no current consensus on antibiotic duration for childhood osteomyelitis or septic arthritis.^72–74^ The major guidelines generally recommended a short course of IV antibiotic treatment followed by an oral antimicrobial therapy for 2–3 weeks in septic arthritis and 3 weeks in osteomyelitis.^84^ However, for MRSA or Panton–Valentine leucocidin-producing *Staphylococcus aureus*, 4–6 weeks of treatment are recommended.^72,83^

Most trials that evaluated antibiotic duration for childhood bone and joint infections were underpowered.^85^ These trial results may have limited generalizability as the studies mainly enrolled mild cases with minimal complications, with infections predominantly caused by bacterial pathogens that responded quickly to antibiotic therapy e.g. MSSA.^86^

#### Bloodstream infections

Five trials evaluated antibiotic duration in Gram-negative bloodstream infections. Three trials were performed in adults, and two in neonates. In adult uncomplicated Gram-negative bloodstream infections, 7 days of treatment was non-inferior to 14 days of antibiotic treatment in terms of clinical failure using a 10% non-inferiority margin.^87,88^ The interpretation of one trial was limited by very low primary outcome rate (5% 30 day clinical failure) compared with the 10% non-inferiority margin.^88^ As for the other trial, its results could only be applied to Enterobacterales bloodstream infections (over 90% of patients enrolled), and in patients whose infection sources were controlled.^87^ In neonates, 10 days was comparable to 14 days if the infant clinically improved and C-reactive protein normalized.^89,90^ However, the findings might not be generalizable as both studies were conducted in single centres in India and sample sizes were small (202 patients in total). There was no randomized trial evidence to guide antibiotic duration for MDR Gram-negative bloodstream infections or for immunocompromised patients.

There was one randomized trial that studied antibiotic duration in *S. aureus* bloodstream infection. In this trial, the majority of the patients enrolled had CoNS bloodstream infections.^91^ The current guidelines recommended a conservative approach of at least 2 weeks of IV antibiotics, with extension to more than 4 weeks if clinical or microbiological response was delayed, in the presence of prothesis or secondary sites of dissemination.^92^

In bloodstream infections complicated by infective endocarditis, international guidelines typically recommended antibiotic duration of up to 6 weeks. This was established empirically with no randomized controlled trial data.^93–96^

#### Non-tuberculous meningitis

Seven trials evaluated antibiotic duration for non-tuberculous meningitis in neonates and children. Antibiotic durations vary according to bacterial pathogen, and should be determined according to clinical response and microbiological clearance (Table 1). Although a 2009 meta-analysis found that 4–7 days was similar to 7–14 days in terms of clinical cure rate, it also highlighted important methodological issues including lack of blinding, small sample sizes, relatively short follow-up and, most importantly, high variability in the causative pathogens.^97^ None of the trials evaluated antibiotic duration in adult meningitis.

#### Cellulitis and skin abscesses

There were 10 trials that studied antibiotic duration for cellulitis and skin abscess. In uncomplicated cellulitis, four trials showed that 5–6 days of oral antibiotics was adequate, if clinical response was observed at the end of treatment.^98–101^ Single doses of dalbavancin,^102^ azithromycin^103^ or oritavancin were also non-inferior to longer courses, including for skin and soft tissue infections caused by MRSA.^104^ However, in these trials, definition of clinical response was poorly defined and was at the discretion of the treating physician.^105^ There were no trials that compared antibiotic duration for complicated soft tissue infections, e.g. recurrent cellulitis or abscesses.

#### Intra-abdominal infections

Nine trials studied antibiotic duration in intraabdominal infections. Generally, about 4 days was comparable to longer courses given adequate surgical control of infection source.^106,107^ The landmark trial that established this compared a 4 day course versus a maximum 10 day course, which found comparable surgical site infection, recurrent intra-abdominal infection, or death (56/257; 22% in the 4 day group versus 58/260; 22%, difference −0.5%, 95% CI −7.0 to 8.0, *P* = 0.92).^107^

There were no trials that compared antibiotic duration for intra-abdominal infections with inadequate source control, and deep organ abscesses. However, adequacy of source control, infection sites, bacterial pathogens and antibiotic resistance patterns are highly heterogeneous and challenging to define.

#### Perioperative prophylaxis

Perioperative prophylaxis is the most well-studied indication, with 52 antibiotic duration trials. Generally, a single dose or repeated dosing for less than 24 h was found to be adequate, across different surgical procedures. This was associated with comparable efficacy at preventing surgical site infections and fewer antibiotic side effects.^108–114^ For some procedures in a clean surgical field without prosthesis insertion, such as head and neck operations and orthopaedic procedures, IDSA did not recommend any antibiotic prophylaxis.^12,71,115–117^

Evidence gaps remain in defining redosing frequencies, especially in certain specialized types of surgery, e.g. cardiothoracic surgery, where surgery may be complicated and prolonged.^115^

### Quality of trial design, conduct and analysis

#### Bias assessment

Bias assessment with the RoB 2 tool was performed for 80 trials (25%), which were published from 2016 onwards and were adequately powered. Twenty-seven (34%), 41 (51%) and 12 (15%) trials were assessed to be at high risk, with some concerns, and low risk of bias, respectively (Supplementary material). The studies classified as having the highest risk of bias were those that were unblinded, had a high degree of non-adherence or crossovers (Figure S1) and those where the analysis failed to account adequately for non-adherence. Ninety-five (30%) of the trials were double-blinded.

Out of 89 non-inferiority or equivalence trials published since 2006, 62 trials reported on the number of adherent participants. The majority of these trials had non-adherence (90%; 56/62), with a median of 11% non-adherent participants (IQR 5%–19%). Only 17 trials reported both the prespecified and actual treatment durations observed in the respective randomization arms; the difference between the actual and the intended treatment durations stated in the protocol ranged from −3 to 6 days (mean of 0 days).

Most of the trials published since 2006 reported a clinical outcome (97%; 143/147). The overall mortality reported in the trials was low (median 0%, IQR 0%–2%). Sixty-seven trials (46%; 67/147) obtained follow-up samples from the participants to check for clearance or newly acquired antibiotic-resistant bacteria at the sites of infection. Only 17 trials (12%) took surveillance cultures during follow-up to assess for emergence of resistant bacteria colonization, most commonly in the upper respiratory, urinary or gastrointestinal tracts. Additionally, 43% (63/147) of the trials reported economic outcomes, including lengths of stay, direct costs (of antibiotics) or indirect costs (consumables and manpower to deliver antibiotics).

#### Non-inferiority margin

Of the 86 non-inferiority trials, 76 reported a non-inferiority margin (88%). The mean non-inferiority margin was 10%, ranging from 0.35% to 25% (Figure S2).

## Discussion

In this systematic review of antibiotic duration trials published to date, most trials (87%; 274/315) successfully concluded that shorter antibiotic courses had been comparable or non-inferior to longer courses. These trials mostly studied infections associated with low mortality, such as respiratory tract and genitourinary infections, and perioperative prophylaxis. Patients from low- and middle-income countries, critically ill patients and children were under-represented. Using arbitrarily determined duration was the most common trial intervention throughout the years. Non-adherence to allocated antibiotic duration was prevalent and involved a median proportion of 11% in each trial. Few trials took surveillance cultures to assess for resistant bacteria colonization with various antibiotic durations.

Some infections had high-quality evidence to shorten antibiotic duration, such as community-acquired pneumonia and uncomplicated urinary tract infections. However, there are also conditions in which practice guidelines have continued to support long treatment duration despite numerous randomized trials showing non-inferiority of a short course in terms of clinical outcomes. These include infections that require longer follow-up (e.g. immunological sequelae of GAS infections), those in low- and middle-income countries where antimicrobial resistance to first-line empirical antibiotics is prevalent (e.g. otitis media in children), and highly heterogeneous infections for which trials with broad inclusion criteria were not informative but where, if inclusion criteria are too specific, participant accruement becomes challenging (e.g. bacterial meningitis, prosthetic joint infections). There remain few randomized trials defining antibiotic duration for relatively common conditions such as Gram-negative and *S. aureus* bloodstream infections and ventilator-associated pneumonia caused by non-fermenting Gram-negative bacilli.

There is limited evidence to show the effectiveness of shortening antibiotic duration in reducing AMR. The effect of antibiotic duration on the development and spread of AMR is expected to vary due to multiple pathogens, host and environmental factors. The penalties of prolonged antibiotic courses are the side effects and associated costs for both treated individuals and, through increased selection for resistance, the population. Direct side effects of antibiotics for the individuals include allergies, kidney and liver injuries, and opportunistic infections such as *Clostridioides difficile* colitis. Though mostly mild, these side effects are not infrequent and have been reported in up to 20% of hospitalized patients who received antibiotics.^118,119^ While there are sound theoretical reasons for expecting reduced antibiotic duration to often lead to reduced AMR, surprisingly little empirical evidence has been collected to explore this relationship. As noted in this review, most antibiotic duration trials investigated the short-term clinical outcomes of reduced antibiotic duration on individual patients, but only nine followed up treated patients to determine subsequent AMR colonization. This is echoed by earlier reviews of studies attempting to quantify the impact of stewardship strategies, which found that microbiology outcomes are seldom reported, especially those from longer-term follow-up.^3^

Non-inferiority was the most commonly adopted design for antibiotic duration trials. Non-inferiority trials compare the short with the standard treatment duration against a margin that the investigators are willing to sacrifice in terms of treatment effects, to reap benefits such as reduced resistance, cost savings or reduced side effects. The non-inferiority margin is usually defined with prior knowledge of the standard-of-care antibiotic duration’s efficacy compared with a placebo, and consensus from subject-matter experts.^120^ However, prior placebo-controlled trials to inform the choice of the non-inferiority margin are frequently lacking, and the efficacy of standard-of-care antibiotic duration may be increasing with time due to improvements in general healthcare delivery.^121^ This systematic review showed that 86% of the antibiotic duration non-inferiority trials published in the last 18 years reported a low event rate of less than 10%. A lower-than-expected event rate can lead to insufficient power, favouring non-inferiority.^122^

## Conclusions

Reducing antibiotic duration is likely to remain an important strategy for antibiotic stewardship, and an area of active research. Innovative and robust trial designs have been proposed to define duration–response relationships, rather than relying on arbitrary durations compared with non-inferiority hypotheses.^123^ Larger randomized trials will allow for meaningful conclusions for patient subsets with varying host and pathogen characteristics. More evidence to define treatment duration is needed for severe bacterial infections and in low- and middle-income settings.

## Supplementary Material

dlae215_Supplementary_Data

## Data Availability

Data extracted from all included antibiotic treatment duration randomized controlled trials are available at https://github.com/moyinNUHS/abxdur-review.

## References

[1] Holmes AH, Moore LSP, Sundsfjord A et al Understanding the mechanisms and drivers of antimicrobial resistance. Lancet 2016; 387: 176–87. 10.1016/S0140-6736(15)00473-0 26603922

[2] Dyar OJ, Huttner B, Schouten J et al What is antimicrobial stewardship? Clin Microbiol Infect 2017; 23: 793–8. 10.1016/j.cmi.2017.08.026 28882725

[3] Schweitzer VA, van Heijl I, van Werkhoven CH et al The quality of studies evaluating antimicrobial stewardship interventions: a systematic review. Clin Microbiol Infect 2019; 25: 555–61. 10.1016/j.cmi.2018.11.002 30472426

[4] Rice LB . The Maxwell Finland lecture: for the duration—rational antibiotic administration in an era of antimicrobial resistance and *Clostridium difficile*. Clin Infect Dis 2008; 46: 491–6. 10.1086/526535 18194098

[5] Llewelyn MJ, Fitzpatrick JM, Darwin E et al The antibiotic course has had its day. BMJ 2017; 358: j3418. 10.1136/bmj.j3418 28747365

[6] Nora D, Salluh J, Martin-Loeches I et al Biomarker-guided antibiotic therapy—strengths and limitations. Ann Transl Med 2017; 5: 208. 10.21037/atm.2017.04.04 28603723 PMC5451622

[7] Bramer WM, Rethlefsen ML, Kleijnen J et al Optimal database combinations for literature searches in systematic reviews: a prospective exploratory study. Syst Rev 2017; 6: 245. 10.1186/s13643-017-0644-y 29208034 PMC5718002

[8] Sterne JA, Savović J, Page MJ et al RoB 2: a revised tool for assessing risk of bias in randomised trials. BMJ 2019; 366: l4898. 10.1136/bmj.l4898 31462531

[9] Falagas ME, Vouloumanou EK, Matthaiou DK et al Effectiveness and safety of short-course vs long-course antibiotic therapy for group A β-hemolytic streptococcal tonsillopharyngitis: a meta-analysis of randomized trials. Mayo Clin Proc 2008; 83: 880–9. 18674472

[10] Shulman ST, Bisno AL, Clegg HW et al Clinical practice guideline for the diagnosis and management of group A streptococcal pharyngitis: 2012 update by the Infectious Diseases Society of America. Clin Infect Dis 2012; 55: e86–102. 10.1093/cid/cis629 22965026 PMC7108032

[11] NICE . Sore throat (acute): antimicrobial prescribing, recommendations. NG84. 2018. https://www.nice.org.uk/guidance/ng84/chapter/Recommendations#choice-of-antibiotic.

[12] WHO . The WHO AWaRe (Access, Watch, Reserve) antibiotic book. 2022. https://www.who.int/publications-detail-redirect/9789240062382.

[13] Tack KJ, Henry DC, Gooch WM et al Five-day cefdinir treatment for streptococcal pharyngitis. Antimicrob Agents Chemother 1998; 42: 1073–5. 10.1128/AAC.42.5.1073 9593129 PMC105747

[14] Casey JR, Pichichero ME. Higher dosages of azithromycin are more effective in treatment of group A streptococcal tonsillopharyngitis. Clin Infect Dis 2005; 40: 1748–55. 10.1086/430307 15909262

[15] Holm AE, Llor C, Bjerrum L et al Short- vs. long-course antibiotic treatment for acute streptococcal pharyngitis: systematic review and meta-analysis of randomized controlled trials. Antibiotics (Basel) 2020; 9: 733. 10.3390/antibiotics9110733 33114471 PMC7692631

[16] Hoberman A, Paradise JL, Rockette HE et al Shortened antimicrobial treatment for acute otitis media in young children. N Engl J Med 2016; 375: 2446–56. 10.1056/NEJMoa1606043 28002709 PMC5319589

[17] Arguedas A, Soley C, Kamicker BJ et al Single-dose extended-release azithromycin versus a 10-day regimen of amoxicillin/clavulanate for the treatment of children with acute otitis media. Int J Infect Dis 2011; 15: e240–8. 10.1016/j.ijid.2010.12.003 21269858

[18] Cohen R, Levy C, Boucherat M et al A multicenter, randomized, double-blind trial of 5 versus 10 days of antibiotic therapy for acute otitis media in young children. J Pediatr 1998; 133: 634–9. 10.1016/S0022-3476(98)70103-9 9821420

[19] Hoberman A, Paradise JL, Burch DJ et al Equivalent efficacy and reduced occurrence of diarrhea from a new formulation of amoxicillin/clavulanate potassium (Augmentin^®^) for treatment of acute otitis media in children. Pediatr Infect Dis J 1997; 16: 463–70. 10.1097/00006454-199705000-00002 9154538

[20] Lieberthal AS, Carroll AE, Chonmaitree T et al The diagnosis and management of acute otitis media. Pediatrics 2013; 131: e964–99. 10.1542/peds.2012-3488 23439909

[21] NICE . Otitis media (acute): antimicrobial prescribing, Recommendations. NG91. 2018. https://www.nice.org.uk/guidance/ng91/chapter/recommendations#children-and-young-people-who-may-be-less-likely-to-benefit-from-antibiotics-those-not-covered-by.

[22] Suzuki HG, Dewez JE, Nijman RG et al Clinical practice guidelines for acute otitis media in children: a systematic review and appraisal of European national guidelines. BMJ Open 2020; 10: e035343. 10.1136/bmjopen-2019-035343 PMC7228535 32371515

[23] Kenna MA . Acute Otitis Media—The Long and the Short of It. 2016. http://www.nejm.org/doi/full/10.1056/NEJMe1614712. 10.1056/NEJMe1614712 28002710

[24] Falagas ME, Karageorgopoulos DE, Grammatikos AP et al Effectiveness and safety of short vs. long duration of antibiotic therapy for acute bacterial sinusitis: a meta-analysis of randomized trials. Br J Clin Pharmacol 2009; 67: 161–71. 10.1111/j.1365-2125.2008.03306.x 19154447 PMC2670373

[25] Wald ER, Applegate KE, Bordley C et al Clinical practice guideline for the diagnosis and management of acute bacterial sinusitis in children aged 1 to 18 years. Pediatrics 2013; 132: e262–80. 10.1542/peds.2013-1071 23796742

[26] Chow AW, Benninger MS, Brook I et al IDSA clinical practice guideline for acute bacterial rhinosinusitis in children and adults. Clin Infect Dis 2012; 54: e72–112. 10.1093/cid/cis370 22438350

[27] NICE . Sinusitis (acute): antimicrobial prescribing, Recommendations NG79. 2017. https://www.nice.org.uk/guidance/ng79/chapter/recommendations.

[28] Li JZ, Winston LG, Moore DH et al Efficacy of short-course antibiotic regimens for community-acquired pneumonia: a meta-analysis. Am J Med 2007; 120: 783–90. 10.1016/j.amjmed.2007.04.023 17765048

[29] el Moussaoui R, de Borgie CAJM, van den Broek P et al Effectiveness of discontinuing antibiotic treatment after three days versus eight days in mild to moderate-severe community acquired pneumonia: randomised, double blind study. BMJ 2006; 332: 1355. 10.1136/bmj.332.7554.1355 16763247 PMC1479094

[30] Haider BA, Saeed MA, Bhutta ZA. Short-course versus long-course antibiotic therapy for non-severe community-acquired pneumonia in children aged 2 months to 59 months. Cochrane Database Syst Rev 2008; issue 4: CD005976. 18425930 10.1002/14651858.CD005976.pub2

[31] Greenberg D, Givon-Lavi N, Sadaka Y et al Short-course antibiotic treatment for community-acquired alveolar pneumonia in ambulatory children: a double-blind, randomized, placebo-controlled trial. Pediatr Infect Dis J 2014; 33: 136–42. 10.1097/INF.0000000000000023 23989106

[32] NICE . Pneumonia (community-acquired): antimicrobial prescribing, Recommendations NG138. 2019. https://www.nice.org.uk/guidance/ng138/chapter/Recommendations.

[33] Tansarli GS, Mylonakis E. Systematic review and meta-analysis of the efficacy of short-course antibiotic treatments for community-acquired pneumonia in adults. Antimicrob Agents Chemother 2018; 62: e00635-18. 10.1128/AAC.00635-18 29987137 PMC6125522

[34] Bradley JS, Byington CL, Shah SS et al The management of community-acquired pneumonia in infants and children older than 3 months of age: clinical practice guidelines by the Pediatric Infectious Diseases Society and the Infectious Diseases Society of America. Clin Infect Dis 2011; 53: e25–76. 10.1093/cid/cir531 21880587 PMC7107838

[35] Martin-Loeches I, Torres A, Nagavci B et al ERS/ESICM/ESCMID/ALAT guidelines for the management of severe community-acquired pneumonia. Intensive Care Med 2023; 49: 615–32. 10.1007/s00134-023-07033-8 37012484 PMC10069946

[36] Woodhead M, Blasi F, Ewig S et al Guidelines for the management of adult lower respiratory tract infections - full version. Clin Microbiol Infect 2011; 17: E1–59. 10.1111/j.1469-0691.2011.03602.x PMC7128977 21951385

[37] López-Alcalde J, Rodriguez-Barrientos R, Redondo-Sánchez J et al Short-course versus long-course therapy of the same antibiotic for community-acquired pneumonia in adolescent and adult outpatients. Cochrane Database Syst Rev 2018; issue 9: CD009070. 10.1002/14651858.CD009070.pub2 30188565 PMC6513237

[38] Kuitunen I, Jääskeläinen J, Korppi M et al Antibiotic treatment duration for community-acquired pneumonia in outpatient children in high-income countries—a systematic review and meta-analysis. Clin Infect Dis 2023; 76: e1123–8. 10.1093/cid/ciac374 35579504 PMC9907524

[39] Bielicki JA, Stöhr W, Barratt S et al Effect of amoxicillin dose and treatment duration on the need for antibiotic re-treatment in children with community-acquired pneumonia. JAMA 2021; 326: 1713–24. 10.1001/jama.2021.17843 34726708 PMC8564579

[40] Williams DJ, Creech CB, Walter EB et al Short- vs standard-course outpatient antibiotic therapy for community-acquired pneumonia in children. JAMA Pediatr 2022; 176: 253–61. 10.1001/jamapediatrics.2021.5547 35040920 PMC8767493

[41] Ginsburg A-S, Mvalo T, Nkwopara E et al Amoxicillin for 3 or 5 days for chest-indrawing pneumonia in Malawian children. N Engl J Med 2020; 383: 13–23. 10.1056/NEJMoa1912400 32609979 PMC7233470

[42] Chang AB, Grimwood K. Antibiotics for childhood pneumonia—do we really know how long to treat? N Engl J Med 2020; 383: 77–9. 10.1056/NEJMe2016328 32609987

[43] Mazlan MZ, Ismail MA, Ali S et al Efficacy and safety of the point-of-care procalcitonin test for determining the antibiotic treatment duration in patients with ventilator-associated pneumonia in the intensive care unit: a randomised controlled trial. Anaesthesiol Intensive Ther 2021: 53: 207–14. 10.5114/ait.2021.104300 34006044 PMC10158486

[44] Fekih Hassen M, Ayed S, Ben Sik Ali H et al Durée de l’antibiothérapie lors du traitement des pneumopathies acquises sous ventilation mécanique: comparaison entre sept jours et dix jours. Étude pilote. Ann Fr Anesth Réanim 2009; 28: 16–23. 10.1016/j.annfar.2008.10.021 19097848

[45] Stolz D, Smyrnios N, Eggimann P et al Procalcitonin for reduced antibiotic exposure in ventilator-associated pneumonia: a randomised study. Eur Respir J 2009; 34: 1364–75. 10.1183/09031936.00053209 19797133

[46] Micek ST, Ward S, Fraser VJ et al A randomized controlled trial of an antibiotic discontinuation policy for clinically suspected ventilator-associated pneumonia. Chest 2004; 125: 1791–9. 10.1378/chest.125.5.1791 15136392

[47] Chastre J, Wolff M, Fagon J-Y et al Comparison of 8 vs 15 days of antibiotic therapy for ventilator-associated pneumonia in adults: a randomized trial. JAMA 2003; 290: 2588–98. 10.1001/jama.290.19.2588 14625336

[48] Mo Y, Booraphun S, Li AY et al Individualised, short-course antibiotic treatment versus usual long-course treatment for ventilator-associated pneumonia (REGARD-VAP): a multicentre, individually randomised, open-label, non-inferiority trial. Lancet Respir Med 2024; 12: 399–408. 10.1016/S2213-2600(23)00418-6 38272050

[49] Torres A, Niederman MS, Chastre J et al International ERS/ESICM/ESCMID/ALAT guidelines for the management of hospital-acquired pneumonia and ventilator-associated pneumonia: guidelines for the management of hospital-acquired pneumonia (HAP)/ventilator-associated pneumonia (VAP) of the European Respiratory Society (ERS), European Society of Intensive Care Medicine (ESICM), European Society of Clinical Microbiology and Infectious Diseases (ESCMID) and Asociación Latinoamericana del Tórax (ALAT). Eur Respir J 2017; 50: 1700582. 10.1183/13993003.00582-2017 28890434

[50] Kalil AC, Metersky ML, Klompas M et al Management of adults with hospital-acquired and ventilator-associated pneumonia: 2016 clinical practice guidelines by the Infectious Diseases Society of America and the American Thoracic Society. Clin Infect Dis 2016; 63: e61–111. 10.1093/cid/ciw353 27418577 PMC4981759

[51] Pugh R, Grant C, Cooke RPD et al Short-course versus prolonged-course antibiotic therapy for hospital-acquired pneumonia in critically ill adults. Cochrane Database Syst Rev 2015; issue 8: CD007577. 26301604 10.1002/14651858.CD007577.pub3 PMC7025798

[52] Bouglé A, Tuffet S, Federici L et al Comparison of 8 versus 15 days of antibiotic therapy for *Pseudomonas aeruginosa* ventilator-associated pneumonia in adults: a randomized, controlled, open-label trial. Intensive Care Med 2022; 48: 841–9. 10.1007/s00134-022-06690-5 35552788

[53] Schuetz P, Wirz Y, Sager R et al Effect of procalcitonin-guided antibiotic treatment on mortality in acute respiratory infections: a patient level meta-analysis. Lancet Infect Dis 2018; 18: 95–107. 10.1016/S1473-3099(17)30592-3 29037960

[54] Stolbrink M, Amiry J, Blakey JD. Does antibiotic treatment duration affect the outcomes of exacerbations of asthma and COPD? A systematic review. Chron Respir Dis 2018; 15: 225–40. 10.1177/1479972317745734 29232988 PMC6100164

[55] Gupta K, Hooton TM, Naber KG et al International clinical practice guidelines for the treatment of acute uncomplicated cystitis and pyelonephritis in women: a 2010 update by the Infectious Diseases Society of America and the European Society for Microbiology and Infectious Diseases. Clin Infect Dis 2011; 52: e103–20. 10.1093/cid/ciq257 21292654

[56] NICE . Urinary tract infection (lower): antimicrobial prescribing, Recommendations. NG109. 2018. https://www.nice.org.uk/guidance/ng109/chapter/Recommendations.

[57] European Association of Urology. Urological Infections—the Guideline. 2024. https://uroweb.org/guidelines/urological-infections/chapter/the-guideline.

[58] NICE . Urinary tract infection (lower): antimicrobial prescribing, Summary of the evidence. NG109. 2018. https://www.nice.org.uk/guidance/ng109/chapter/Summary-of-the-evidence#choice-of-antibiotic-2.

[59] Drekonja DM, Trautner B, Amundson C et al Effect of 7 vs 14 days of antibiotic therapy on resolution of symptoms among afebrile men with urinary tract infection: a randomized clinical trial. JAMA 2021; 326: 324–31. 10.1001/jama.2021.9899 34313686 PMC8317010

[60] Lafaurie M, Chevret S, Fontaine J-P et al Antimicrobial for 7 or 14 days for febrile urinary tract infection in men: a multicenter noninferiority double-blind, placebo-controlled, randomized clinical trial. Clin Infect Dis 2023; 76: 2154–62. 10.1093/cid/ciad070 36785526

[61] Peterson J, Kaul S, Khashab M et al A double-blind, randomized comparison of levofloxacin 750 mg once-daily for five days with ciprofloxacin 400/500 mg twice-daily for 10 days for the treatment of complicated urinary tract infections and acute pyelonephritis. Urology 2008; 71: 17–22. 10.1016/j.urology.2007.09.002 18242357

[62] Darouiche RO, Mohajer A, Siddiq DM et al Short versus long course of antibiotics for catheter-associated urinary tract infections in patients with spinal cord injury: a randomized controlled noninferiority trial. Arch Phys Med Rehabil 2014; 95: 290–6. 10.1016/j.apmr.2013.09.003 24035770

[63] Raz R, Schiller D, Nicolle LE. Chronic indwelling catheter replacement before antimicrobial therapy for symptomatic urinary tract infection. J Urol 2000; 164: 1254–8. 10.1016/S0022-5347(05)67150-9 10992375

[64] NICE. Urinary tract infection (catheter-associated): antimicrobial prescribing, Recommendations. NG113. 2018. https://www.nice.org.uk/guidance/ng113/chapter/Recommendations.

[65] Hooton TM, Bradley SF, Cardenas DD et al Diagnosis, prevention, and treatment of catheter-associated urinary tract infection in adults: 2009 international clinical practice guidelines from the Infectious Diseases Society of America. Clin Infect Dis 2010; 50: 625–63. 10.1086/650482 20175247

[66] Talan DA, Stamm WE, Hooton TM et al Comparison of ciprofloxacin (7 days) and trimethoprim-sulfamethoxazole (14 days) for acute uncomplicated pyelonephritis in women: a randomized trial. JAMA 2000; 283: 1583–90. 10.1001/jama.283.12.1583 10735395

[67] Bernard L, Arvieux C, Brunschweiler B et al Antibiotic therapy for 6 or 12 weeks for prosthetic joint infection. N Engl J Med 2021; 384: 1991–2001. 10.1056/NEJMoa2020198 34042388

[68] Osmon DR, Berbari EF, Berendt AR et al Diagnosis and management of prosthetic joint infection: clinical practice guidelines by the Infectious Diseases Society of America. Clin Infect Dis 2013; 56: e1–25. 10.1093/cid/cis966 23223583

[69] NICE, BNF. Musculoskeletal system infections, antimicrobial therapy, Treatment summaries. 2020. https://bnf.nice.org.uk/treatment-summaries/musculoskeletal-system-infections-antibacterial-therapy/.

[70] NICE . Joint replacement (primary): hip, knee and shoulder, Recommendations. NG157. 2020. https://www.nice.org.uk/guidance/ng157/chapter/Recommendations. 32881469

[71] NICE . Surgical site infections: prevention and treatment, Recommendations. NG125. 2019. https://www.nice.org.uk/guidance/ng125/chapter/Recommendations.

[72] Liu C, Bayer A, Cosgrove SE et al Clinical practice guidelines by the Infectious Diseases Society of America for the treatment of methicillin-resistant *Staphylococcus aureus* infections in adults and children: executive summary. Clin Infect Dis 2011; 52: 285–92. 10.1093/cid/cir034 21217178

[73] Concia E, Prandini N, Massari L et al Osteomyelitis: clinical update for practical guidelines. Nucl Med Commun 2006; 27: 645–60. 10.1097/00006231-200608000-00007 16829765

[74] Ravn C, Neyt J, Benito N et al Guideline for management of septic arthritis in native joints (SANJO). J Bone Jt Infect 2023; 8: 29–37. 10.5194/jbji-8-29-2023 36756304 PMC9901514

[75] Le Vavasseur B, Zeller V. Antibiotic therapy for prosthetic joint infections: an overview. Antibiotics 2022; 11: 486. 10.3390/antibiotics11040486 35453237 PMC9025623

[76] Malahias M-A, Gu A, Harris EC et al The role of long-term antibiotic suppression in the management of peri-prosthetic joint infections treated with debridement, antibiotics, and implant retention: a systematic review. J Arthroplasty 2020; 35: 1154–60. 10.1016/j.arth.2019.11.026 31955984

[77] Gariani K, Pham T-T, Kressmann B et al Three weeks versus six weeks of antibiotic therapy for diabetic foot osteomyelitis: a prospective, randomized, noninferiority pilot trial. Clin Infect Dis 2021; 73: e1539–45. 10.1093/cid/ciaa1758 33242083

[78] Chu Y, Wang C, Zhang J et al Can we stop antibiotic therapy when signs and symptoms have resolved in diabetic foot infection patients? Int J Low Extrem Wounds 2015; 14: 277–83. 10.1177/1534734615596891 26248828 PMC4601082

[79] Tone A, Nguyen S, Devemy F et al Six-week versus twelve-week antibiotic therapy for nonsurgically treated diabetic foot osteomyelitis: a multicenter open-label controlled randomized study. Diabetes Care 2014; 38: 302–7. 10.2337/dc14-1514 25414157

[80] Bernard L, Dinh A, Ghout I et al Antibiotic treatment for 6 weeks versus 12 weeks in patients with pyogenic vertebral osteomyelitis: an open-label, non-inferiority, randomised, controlled trial. Lancet 2015; 385: 875–82. 10.1016/S0140-6736(14)61233-2 25468170

[81] Gjika E, Beaulieu J-Y, Vakalopoulos K et al Two weeks versus four weeks of antibiotic therapy after surgical drainage for native joint bacterial arthritis: a prospective, randomised, non-inferiority trial. Ann Rheum Dis 2019; 78: 1114–21. 10.1136/annrheumdis-2019-215116 30992295 PMC6691865

[82] Yen H-T, Hsieh RW, Huang C-Y et al Short-course versus long-course antibiotics in prosthetic joint infections: a systematic review and meta-analysis of one randomized controlled trial plus nine observational studies. J Antimicrob Chemother 2019; 74: 2507–16. 10.1093/jac/dkz166 31050758

[83] Peltola H, Pääkkönen M, Kallio P et al Prospective, randomized trial of 10 days versus 30 days of antimicrobial treatment, including a short-term course of parenteral therapy, for childhood septic arthritis. Clin Infect Dis 2009; 48: 1201–121. 10.1086/597582 19323633

[84] Castellazzi L, Mantero M, Esposito S. Update on the management of pediatric acute osteomyelitis and septic arthritis. Int J Mol Sci 2016; 17: 855. 10.3390/ijms17060855 27258258 PMC4926389

[85] Howard-Jones AR, Isaacs D. Systematic review of duration and choice of systemic antibiotic therapy for acute haematogenous bacterial osteomyelitis in children. J Paediatr Child Health 2013; 49: 760–8. 10.1111/jpc.12251 23745943

[86] Peltola H, Pääkkönen M, Kallio P et al Short- versus long-term antimicrobial treatment for acute hematogenous osteomyelitis of childhood: prospective, randomized trial on 131 culture-positive cases. Pediatr Infect Dis J 2010; 29: 1123–8. 10.1097/INF.0b013e3181f55a89 20842069

[87] Yahav D, Franceschini E, Koppel F et al Seven versus 14 days of antibiotic therapy for uncomplicated gram-negative bacteremia: a noninferiority randomized controlled trial. Clin Infect Dis 2019; 69: 1091–8. 10.1093/cid/ciy1054 30535100

[88] von Dach E, Albrich WC, Brunel A-S et al Effect of C-reactive protein–guided antibiotic treatment duration, 7-day treatment, or 14-day treatment on 30-day clinical failure rate in patients with uncomplicated gram-negative bacteremia: a randomized clinical trial. JAMA 2020; 323: 2160–9. 10.1001/jama.2020.6348 32484534 PMC7267846

[89] Reddy A, Sathenahalli V, Shivanna N et al Ten versus 14 days of antibiotic therapy in culture-proven neonatal sepsis: a randomized, controlled trial. Indian J Pediatr 2022; 89: 339–42. 10.1007/s12098-021-03794-6 34097231

[90] Rohatgi S, Dewan P, Faridi MMA et al Seven versus 10 days antibiotic therapy for culture-proven neonatal sepsis: a randomised controlled trial. J Paediatr Child Health 2017; 53: 556–62. 10.1111/jpc.13518 28398692

[91] Holland TL, Raad I, Boucher HW et al Effect of algorithm-based therapy vs usual care on clinical success and serious adverse events in patients with staphylococcal bacteremia: a randomized clinical trial. JAMA 2018; 320: 1249–58. 10.1001/jama.2018.13155 30264119 PMC6233609

[92] Eichenberger EM, Fowler VG, Holland TL. Duration of antibiotic therapy for Staphylococcus aureus bacteraemia: the long and the short of it. Clin Microbiol Infect 2020; 26: 536–8. 10.1016/j.cmi.2020.01.003 31968272 PMC7278270

[93] Delgado V, Ajmone Marsan N, de Waha S et al 2023 ESC guidelines for the management of endocarditis: developed by the task force on the management of endocarditis of the European Society of Cardiology (ESC) endorsed by the European Association for Cardio-Thoracic Surgery (EACTS) and the European Association of Nuclear Medicine (EANM). Eur Heart J 2023; 44: 4780. 10.1093/eurheartj/ehad625 37738322

[94] Baddour LM, Wilson WR, Bayer AS et al Infective endocarditis in adults: diagnosis, antimicrobial therapy, and management of complications. Circulation 2015; 132: 1435–86. 10.1161/CIR.0000000000000296 26373316

[95] Wilson WR, Gewitz M, Lockhart PB et al Prevention of Viridans group streptococcal infective endocarditis: a scientific statement from the American Heart Association. Circulation 2021; 143: e963–78. 10.1161/CIR.0000000000000969 33853363

[96] NICE . Prophylaxis against infective endocarditis: antimicrobial prophylaxis against infective endocarditis in adults and children undergoing interventional procedures, Recommendations. CG64. 2008. https://www.nice.org.uk/guidance/CG64/chapter/Recommendations#infection. 21656971

[97] Karageorgopoulos DE, Valkimadi PE, Kapaskelis A et al Short versus long duration of antibiotic therapy for bacterial meningitis: a meta-analysis of randomised controlled trials in children. Arch Dis Child 2009; 94: 607–14. 10.1136/adc.2008.151563 19628879

[98] Wasilewski MM, Wilson MG, Sides GD et al Comparative efficacy of 5 days of dirithromycin and 7 days of erythromycin in skin and soft tissue infections. J Antimicrob Chemother 2000; 46: 255–62. 10.1093/jac/46.2.255 10933649

[99] Hepburn MJ, Dooley DP, Skidmore PJ et al Comparison of short-course (5 days) and standard (10 days) treatment for uncomplicated cellulitis. Arch Intern Med 2004; 164: 1669–74. 10.1001/archinte.164.15.1669 15302637

[100] Cranendonk DR, Opmeer BC, van Agtmael MA et al Antibiotic treatment for 6 days versus 12 days in patients with severe cellulitis: a multicentre randomized, double-blind, placebo-controlled, non-inferiority trial. Clin Microbiol Infect 2020; 26: 606–12. 10.1016/j.cmi.2019.09.019 31618678

[101] Prokocimer P, De Anda C, Fang E et al Tedizolid phosphate vs linezolid for treatment of acute bacterial skin and skin structure infections: the ESTABLISH-1 randomized trial. JAMA 2013; 309: 559–69. 10.1001/jama.2013.241 23403680

[102] Dunne MW, Puttagunta S, Giordano P et al A randomized clinical trial of single-dose versus weekly dalbavancin for treatment of acute bacterial skin and skin structure infection. Clin Infect Dis 2016; 62: 545–51. 10.1093/cid/civ982 26611777 PMC4741365

[103] Dey SK, Das AK, Sen S et al Comparative evaluation of 2 g single dose versus conventional dose azithromycin in uncomplicated skin and skin structure infections. Indian J Pharmacol 2015; 47: 365–9. 10.4103/0253-7613.161254 26288467 PMC4527055

[104] Corey GR, Good S, Jiang H et al Single-dose oritavancin versus 7–10 days of vancomycin in the treatment of gram-positive acute bacterial skin and skin structure infections: the SOLO II noninferiority study. Clin Infect Dis 2015; 60: 254–62. 10.1093/cid/ciu778 25294250

[105] Stevens DL, Bisno AL, Chambers HF et al Practice guidelines for the diagnosis and management of skin and soft tissue infections: 2014 update by the Infectious Diseases Society of America. Clin Infect Dis 2014; 59: e10–52. 10.1093/cid/ciu296 24973422

[106] Solomkin JS, Mazuski JE, Bradley JS et al Diagnosis and management of complicated intra-abdominal infection in adults and children: guidelines by the Surgical Infection Society and the Infectious Diseases Society of America. Surg Infect (Larchmt) 2010; 11: 79–109. 10.1089/sur.2009.9930 20163262

[107] Sawyer RG, Claridge JA, Nathens AB et al Trial of short-course antimicrobial therapy for intraabdominal infection. N Engl J Med 2015; 372: 1996–2005. 10.1056/NEJMoa1411162 25992746 PMC4469182

[108] WHO . Global Guidelines for the Prevention of Surgical Site Infection, 2nd ed. 2018. https://iris.who.int/handle/10665/277399. 30689333

[109] McDonald M, Grabsch E, Marshall C et al Single-versus multiple–dose antimicrobial prophylaxis for major surgery: a systematic review. Aust N Z J Surg 1998; 68: 388–95. 10.1111/j.1445-2197.1998.tb04785.x 9623456

[110] Conte JE, Cohen SN, Roe BB et al Antibiotic prophylaxis and cardiac surgery. Ann Intern Med 1972; 76: 943–9. 10.7326/0003-4819-76-6-943 5027584

[111] Pollard JP, Hughes SP, Scott JE et al Antibiotic prophylaxis in total hip replacement. Br Med J 1979; 1: 707–9. 10.1136/bmj.1.6165.707 373841 PMC1598803

[112] Harbarth S, Samore MH, Lichtenberg D et al Prolonged antibiotic prophylaxis after cardiovascular surgery and its effect on surgical site infections and antimicrobial resistance. Circulation 2000; 101: 2916–21. 10.1161/01.CIR.101.25.2916 10869263

[113] Bernatz JT, Safdar N, Hetzel S et al Antibiotic overuse is a major risk factor for *Clostridium difficile* infection in surgical patients. Infect Control Hosp Epidemiol 2017; 38: 1254–7. 10.1017/ice.2017.158 28756789

[114] Branch-Elliman W, O’Brien W, Strymish J et al Association of duration and type of surgical prophylaxis with antimicrobial-associated adverse events. JAMA Surg 2019; 154: 590–8. 10.1001/jamasurg.2019.0569 31017647 PMC6487902

[115] Bratzler DW, Dellinger EP, Olsen KM et al Clinical practice guidelines for antimicrobial prophylaxis in surgery. Surg Infect (Larchmt) 2013; 14: 73–156. 10.1089/sur.2013.9999 23461695

[116] ECDC . Peri-operative antimicrobial prophylaxis. 2017. https://www.ecdc.europa.eu/en/publications-data/directory-guidance-prevention-and-control/prudent-use-antibiotics/peri-operative.

[117] Righi E, Mutters NT, Guirao X et al ESCMID/EUCIC clinical practice guidelines on perioperative antibiotic prophylaxis in patients colonized by multidrug-resistant Gram-negative bacteria before surgery. Clin Microbiol Infect 2023; 29: 463–79. 10.1016/j.cmi.2022.12.012 36566836

[118] Tamma PD, Avdic E, Li DX et al Association of adverse events with antibiotic use in hospitalized patients. JAMA Intern Med 2017; 177: 1308–15. 10.1001/jamainternmed.2017.1938 28604925 PMC5710569

[119] Jourdan A, Sangha B, Kim E et al Antibiotic hypersensitivity and adverse reactions: management and implications in clinical practice. Allergy Asthma Clin Immunol 2020; 16: 6. 10.1186/s13223-020-0402-x 31993070 PMC6974965

[120] Schumi J, Wittes JT. Through the looking glass: understanding non-inferiority. Trials 2011; 12: 106. 10.1186/1745-6215-12-106 21539749 PMC3113981

[121] Spellberg B, Talbot GH, Brass EP et al Position paper: Recommended design features of future clinical trials of antibacterial agents for community-acquired pneumonia. Clin Infect Dis 2008; 47: S249–65. 10.1086/591411 19018610 PMC2827629

[122] Kaul S, Diamond GA, Weintraub WS. Trials and tribulations of non-inferiority: the ximelagatran experience. J Am Coll Cardiol 2005; 46: 1986–95. 10.1016/j.jacc.2005.07.062 16325029

[123] Pouwels KB, Yin M, Butler CC et al Optimising trial designs to identify appropriate antibiotic treatment durations. BMC Med 2019; 17: 115. 10.1186/s12916-019-1348-z 31221165 PMC6587258

[124] Chew BH, Reicherz A, Krambeck AE et al Prospective randomized trial of 2 versus 12-weeks of postoperative antibiotics after percutaneous nephrolithotomy in complex patients with infection-related kidney stones. Int J Urol 2022; 29: 1551–58. 10.1111/iju.15045 36102630

[125] de Jong Z, Pontonnier F, Plante P. Single-dose fosfomycin trometamol (Monuril) versus multiple-dose norfloxacin: results of a multicenter study in females with uncomplicated lower urinary tract infections. Urol Int 1991; 46: 344–8. 10.1159/000282164 1926651

[126] Sheehan GJ, Harding GK, Haase DA et al Double-blind, randomized comparison of 24 weeks of norfloxacin and 12 weeks of norfloxacin followed by 12 weeks of placebo in the therapy of complicated urinary tract infection. Antimicrob Agents Chemother 1988; 32: 1292–3. 10.1128/AAC.32.8.1292 3056259 PMC172398

[127] Stansfeld JM . Duration of treatment for urinary tract infections in children. Br Med J 1975; 3: 65–6. 10.1136/bmj.3.5975.65 1095132 PMC1673626

