# Supplementary material for: Antibiotic duration for common bacterial infections—a systematic review
Source: JAC Antimicrob Resist. 2025 Jan 29;7(1):dlae215. doi: 10.1093/jacamr/dlae215 (PMC11775593; doi:10.1093/jacamr/dlae215)
Supplement: dlae215_Supplementary_Data [file dlae215_supplementary_data.zip › Supp_241122.docx]

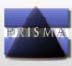
**Supplementary material 1**

**PRISMA 2020 checklist**

| **Section and Topic** | **Item #** | **Checklist item** | **Location where item is reported** |
| --- | --- | --- | --- |
| **TITLE** | | |  |
| Title | 1 | Identify the report as a systematic review. | Cover page |
| **ABSTRACT** | | |  |
| Abstract | 2 | See the PRISMA 2020 for Abstracts checklist. | Abstract section |
| **INTRODUCTION** | | |  |
| Rationale | 3 | Describe the rationale for the review in the context of existing knowledge. | Introduction paragraphs 1-3 |
| Objectives | 4 | Provide an explicit statement of the objective(s) or question(s) the review addresses. | Introduction paragraph 5 |
| **METHODS** | | |  |
| Eligibility criteria | 5 | Specify the inclusion and exclusion criteria for the review and how studies were grouped for the syntheses. | Methods subsection "Study Selection” |
| Information sources | 6 | Specify all databases, registers, websites, organisations, reference lists and other sources searched or consulted to identify studies. Specify the date when each source was last searched or consulted. | Methods subsection "Data Sources and Searches*”* |
| Search strategy | 7 | Present the full search strategies for all databases, registers and websites, including any filters and limits used. | Supplementary material 2 Table S1 |
| Selection process | 8 | Specify the methods used to decide whether a study met the inclusion criteria of the review, including how many reviewers screened each record and each report retrieved, whether they worked independently, and if applicable, details of automation tools used in the process. | Methods subsection "*Study Selection"* |
| Data collection process | 9 | Specify the methods used to collect data from reports, including how many reviewers collected data from each report, whether they worked independently, any processes for obtaining or confirming data from study investigators, and if applicable, details of automation tools used in the process. | Methods subsection "*Data Extraction and Quality Assessment"* |
| Data items | 10a | List and define all outcomes for which data were sought. Specify whether all results that were compatible with each outcome domain in each study were sought (e.g. for all measures, time points, analyses), and if not, the methods used to decide which results to collect. | Methods subsection "*Data Extraction and Quality Assessment "* |
|  | 10b | List and define all other variables for which data were sought (e.g. participant and intervention characteristics, funding sources). Describe any assumptions made about any missing or unclear information. | Methods subsection "*Data Extraction and Quality Assessment "* |
| Study risk of bias assessment | 11 | Specify the methods used to assess risk of bias in the included studies, including details of the tool(s) used, how many reviewers assessed each study and whether they worked independently, and if applicable, details of automation tools used in the process. | Methods subsection " *Data Extraction and Quality Assessment " and* Acknowledgement |
| Effect measures | 12 | Specify for each outcome the effect measure(s) (e.g. risk ratio, mean difference) used in the synthesis or presentation of results. | Methods subsection " *Data Synthesis and Analysis"* |
| Synthesis methods | 13a | Describe the processes used to decide which studies were eligible for each synthesis (e.g. tabulating the study intervention characteristics and comparing against the planned groups for each synthesis (item #5)). | Methods "*Data Synthesis and Analysis"* |
|  | 13b | Describe any methods required to prepare the data for presentation or synthesis, such as handling of missing summary statistics, or data conversions. | Methods "*Data Synthesis and Analysis"* |
|  | 13c | Describe any methods used to tabulate or visually display results of individual studies and syntheses. | Methods "*Data Synthesis and Analysis"* |
|  | 13d | Describe any methods used to synthesize results and provide a rationale for the choice(s). If meta-analysis was performed, describe the model(s), method(s) to identify the presence and extent of statistical heterogeneity, and software package(s) used. | Methods "*Data Synthesis and Analysis"* |
|  | 13e | Describe any methods used to explore possible causes of heterogeneity among study results (e.g. subgroup analysis, meta-regression). | NA |
|  | 13f | Describe any sensitivity analyses conducted to assess robustness of the synthesized results. | NA |
| Reporting bias assessment | 14 | Describe any methods used to assess risk of bias due to missing results in a synthesis (arising from reporting biases). | Methods subsection "*Data Extraction and Quality Assessment"* |
| Certainty assessment | 15 | Describe any methods used to assess certainty (or confidence) in the body of evidence for an outcome. | Methods subsection "*Data Extraction and Quality Assessment"* |
| **RESULTS** | | |  |
| Study selection | 16a | Describe the results of the search and selection process, from the number of records identified in the search to the number of studies included in the review, ideally using a flow diagram. | Results paragraph 1, Figure 1 |
|  | 16b | Cite studies that might appear to meet the inclusion criteria, but which were excluded, and explain why they were excluded. | Results paragraph 1, Figure 1 |
| Study characteristics | 17 | Cite each included study and present its characteristics. | Results subsection "*Overview of antibiotic treatment duration randomised trials"*, Figures 2-4 |
| Risk of bias in studies | 18 | Present assessments of risk of bias for each included study. | Supplementary Material 3 |
| Results of individual studies | 19 | For all outcomes, present, for each study: (a) summary statistics for each group (where appropriate) and (b) an effect estimate and its precision (e.g. confidence/credible interval), ideally using structured tables or plots. | Results subsection "*Overview of antibiotic treatment duration randomised trials",* Table 1, Figures 2-4, Supplementary Material 4 |
| Results of syntheses | 20a | For each synthesis, briefly summarise the characteristics and risk of bias among contributing studies. | Results subsection "*Overview of antibiotic treatment duration randomised trials"* and *“Quality of trial design, conduct and analysis"*, Table 1, Figures 2-4, Supplementary Material 3 and 4 |
|  | 20b | Present results of all statistical syntheses conducted. If meta-analysis was done, present for each the summary estimate and its precision (e.g. confidence/credible interval) and measures of statistical heterogeneity. If comparing groups, describe the direction of the effect. | NA |
|  | 20c | Present results of all investigations of possible causes of heterogeneity among study results. | Results subsection "*Quality of trial design, conduct and analysis”* |
|  | 20d | Present results of all sensitivity analyses conducted to assess the robustness of the synthesized results. | NA |
| Reporting biases | 21 | Present assessments of risk of bias due to missing results (arising from reporting biases) for each synthesis assessed. | Results subsection "*Quality of trial design, conduct and analysis",* Supplementary Material 3 |
| Certainty of evidence | 22 | Present assessments of certainty (or confidence) in the body of evidence for each outcome assessed. | NA |
| **DISCUSSION** | | |  |
| Discussion | 23a | Provide a general interpretation of the results in the context of other evidence. | Discussion paragraphs 1-2 |
|  | 23b | Discuss any limitations of the evidence included in the review. | Discussion paragraphs 3-4 |
|  | 23c | Discuss any limitations of the review processes used. | Discussion paragraph 3-4 |
|  | 23d | Discuss implications of the results for practice, policy, and future research. | Discussion paragraphs 4-5 |
| **OTHER INFORMATION** | | |  |
| Registration and protocol | 24a | Provide registration information for the review, including register name and registration number, or state that the review was not registered. | Methods subsection "Data Sources and Searches*”* |
|  | 24b | Indicate where the review protocol can be accessed, or state that a protocol was not prepared. | Methods subsection "Data Sources and Searches*”* |
|  | 24c | Describe and explain any amendments to information provided at registration or in the protocol. | NA |
| Support | 25 | Describe sources of financial or non-financial support for the review, and the role of the funders or sponsors in the review. | Methods subsection “*Role of the Funding Source”* and Funding section |
| Competing interests | 26 | Declare any competing interests of review authors. | Conflict of Interest sections |
| Availability of data, code and other materials | 27 | Report which of the following are publicly available and where they can be found: template data collection forms; data extracted from included studies; data used for all analyses; analytic code; any other materials used in the review. | Acknowledgement (Data sharing) |

*From:*  Page MJ, McKenzie JE, Bossuyt PM, Boutron I, Hoffmann TC, Mulrow CD, et al. The PRISMA 2020 statement: an updated guideline for reporting systematic reviews. BMJ 2021;372:n71. doi: 10.1136/bmj.n71

For more information, visit: <http://www.prisma-statement.org/>

**Supplementary material 2**

**Table S1: Search terms used in the literature review.**

| **Database** | **Search Term** |
| --- | --- |
| MEDLINE | ((antibiotic) AND (infection)) AND (weeks[Title])  **Filters:** Randomized Controlled Trial, Humans, 1920–2024  **Details:** (((((((("anti bacterial agents"[Pharmacological Action] OR "anti-bacterial agents"  [MeSH Terms]) OR ("anti bacterial"[All Fields] AND "agents"[All Fields])) OR "anti bacterial  agents"[All Fields]) OR "antibiotic"[All Fields]) OR "antibiotics"[All Fields]) OR  antibiotics"[All Fields]) OR "antibiotical"[All Fields]) AND ((((((((((((((((((((("infect" [All Fields] OR "infectability" [All Fields]) OR "infectable"[All Fields]) OR "infectant"[All Fields]) OR "infectants"[All Fields]) OR "infected"[All Fields]) OR "infecteds" [All Fields]) OR "infectibility"[All Fields]) OR "infectible" [All Fields]) OR "infecting"[All Fields]) OR "infections" [All Fields]) OR "infections"[MeSH Terms]) OR "infections"[All Fields]) OR "infection"[All Fields]) OR "infective"[All Fields]) OR "infectiveness" [All Fields]) OR "infectives"[All Fields]) OR "infectivities"[All Fields]) OR "infects" [All Fields]) OR "pathogenicity"[MeSH Subheading]) OR "pathogenicity"[All Fields]) OR "infectivity" [All Fields])) AND "weeks"[Title] |
|  | ((antibiotic) AND (infection)) AND (days[Title])  **Filters:** Randomized Controlled Trial, Humans, 1920–2024  **Details:** (((((((("anti bacterial agents"[Pharmacological Action] OR "anti-bacterial agents"  [MeSH Terms]) OR ("anti bacterial"[All Fields] AND "agents"[All Fields])) OR "anti bacterial  agents"[All Fields]) OR "antibiotic"[All Fields]) OR "antibiotics"[All Fields]) OR  antibiotics"[All Fields]) OR "antibiotical"[All Fields]) AND ((((((((((((((((((((("infect" [All Fields] OR "infectability" [All Fields]) OR "infectable"[All Fields]) OR "infectant"[All Fields]) OR "infectants"[All Fields]) OR "infected"[All Fields]) OR "infecteds" [All Fields]) OR "infectibility"[All Fields]) OR "infectible" [All Fields]) OR "infecting"[All Fields]) OR "infections" [All Fields]) OR "infections"[MeSH Terms]) OR "infections"[All Fields]) OR "infection"[All Fields]) OR "infective"[All Fields]) OR "infectiveness" [All Fields]) OR "infectives"[All Fields]) OR "infectivities"[All Fields]) OR "infects"[All Fields]) OR "pathogenicity"[MeSH Subheading]) OR "pathogenicity"[All Fields]) OR "infectivity"  [All Fields])) AND "days"[Title] |
|  | ((antibiotic) AND (infection)) AND (duration)  **Filters:** Randomized Controlled Trial, Humans, 1920–2024  **Details:** (((((((("anti bacterial agents"[Pharmacological Action] OR "anti-bacterial agents"[MeSH Terms]) OR ("anti bacterial"[All Fields] AND "agents"[All Fields])) OR "anti bacterial agents"[All Fields]) OR "antibiotic"[All Fields]) OR "antibiotics"[All Fields]) OR "antibiotic s"[All Fields]) OR "antibiotical"[All Fields]) AND ((((((((((((((((((((("infect"[All Fields] OR "infectability"[All Fields]) OR "infectable"[All Fields]) OR "infectant"[All Fields]) OR "infectants"[All Fields]) OR "infected"[All Fields]) OR "infecteds" [All Fields]) OR "infectibility"[All Fields]) OR "infectible"[All Fields]) OR "infecting"[All Fields]) OR "infections"[All Fields]) OR "infections"[MeSH Terms]) OR "infections"[All Fields]) OR "infection"[All Fields]) OR "infective"[All Fields]) OR "infectiveness"[All Fields]) OR "infectives"[All Fields]) OR "infectivities"[All Fields]) OR "infects"[All Fields]) OR "pathogenicity"[MeSH Subheading]) OR "pathogenicity"[All Fields]) OR "infectivity"[All Fields])) AND ("duration"[All Fields] OR "durations"  [All Fields]) |
| EMBASE | antibiotic AND infection AND week*:ti AND [randomized controlled trial]/lim AND [1920–2024]/py  AND ’human’/de |
|  | antibiotic AND infection AND day*:ti AND [randomized controlled trial]/lim AND [1920–2024]/py  AND ’human’/de |
|  | antibiotic AND infection AND duration AND [randomized controlled trial]/lim AND [1920–2024]/py  AND ’human’/de |

**Supplementary material 3**

**Risk of Bias Tool 2 assessment**

| Domains | | Assessment (n, % out of total 80 trials*) | | | Justifications for having some concerns or high risk of bias during assessment |
| --- | --- | --- | --- | --- | --- |
|  |  | High risk | Some concerns | Low risk |  |
| 1 | Risk of bias arising from the randomisation process | 0 (0) | 3 (4) | 77 (95) | Unbalanced baseline patient characteristics unlikely due to chance alone |
| 2 | Risk of bias due to deviations from the intended interventions (effect of assignment to intervention or effect of adhering to intervention) | 18 (24) | 42 (53) | 20 (25) | Most trials were unblinded and having acknowledge of the trial interventions may alter subsequent patient care, e.g. more attention paid to patients randomised to short duration arm  Many trials had non-adherence resulting in crossing over of participants, i.e. patients randomised to the short arm actually receiving longer course of antibiotic and vice versa |
| 3 | Risk of bias due to missing outcome data | 2 (3) | 15 (19) | 63 (79) | Substantial missing data or disproportionate missing data in one randomisation arm versus the other |
| 4 | Risk of bias in measurement of the outcome | 3 (4) | 27 (34) | 50 (63) | Many trials did not blind assessors of outcome to the intervention arm |
| 5 | Risk of bias in selection of the reported result | 5 (6) | 24 (30) | 51 (64) | Outcome measures may be subjective, e.g. clinical improvement, without specific definitions  Pre-specified analysis was not available  Multiple analyses could have been performed but not reported, e.g. adjusted versus unadjusted models, conversion of continuous variables to categorical scales |
| Overall | | 27 (34) | 42 (51) | 12 (15) |  |

***** 80 antibiotic duration randomised trials were published in or after 2006, and reported sample size calculations and completed enrolment

**Supplementary material 4**

**Figure S1: Comparison of randomised, analysed and per-protocol trial participants in antibiotic duration trials from 2006 to 2024.** One hundred and fifty-one antibiotic duration randomised trials are presented in the graph. Each horizontal bar represents a unique trial. One trial published with a sample size of more than 3000 participants is not shown on the graph to ensure visibility of the bars plotted from other trials. The green bars on the left represent trial participants who were randomised to the long duration arm; the gold bars on the right represent participants randomised to the short duration arm. The shade of the colours represents different populations of trial participants: the widest bars on each row represent the number of participants randomised in each trial (lightest colours); followed by the number of participants who were reported to be per-protocol; the darkest shades represent the number of participants who crossed over to the opposite arm. Absent bars indicate that the relevant numbers were not reported.


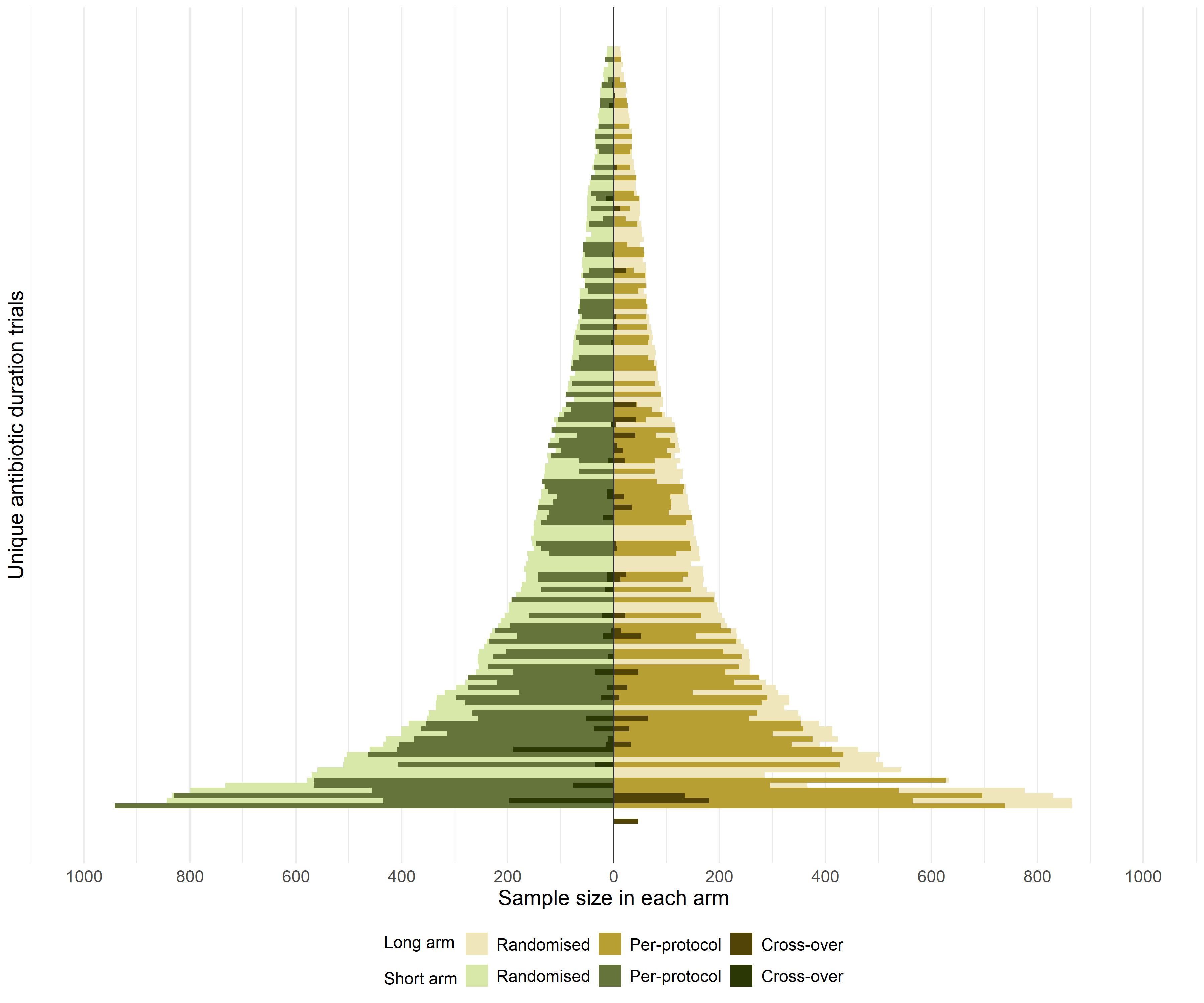


**Figure S2. Trial estimates and respective non-inferiority margins in antibiotic duration non-inferiority trials.** Each vertical line represents a unique comparison between a long and a short duration (absolute difference point estimates and 95% confidence intervals). 147 non-inferiority trials which reported clinical outcomes as the primary outcome are shown. The light-coloured lines represent trial comparisons that did not calculate sample size or did not meet the target sample size. Trial comparisons that failed to conclude non-inferiority are indicated by upper bounds of the vertical error bars crossing the non-inferiority margins (yellow horizontal segments). None of the trials concluded that a short duration was superior to long duration based on the primary outcomes.


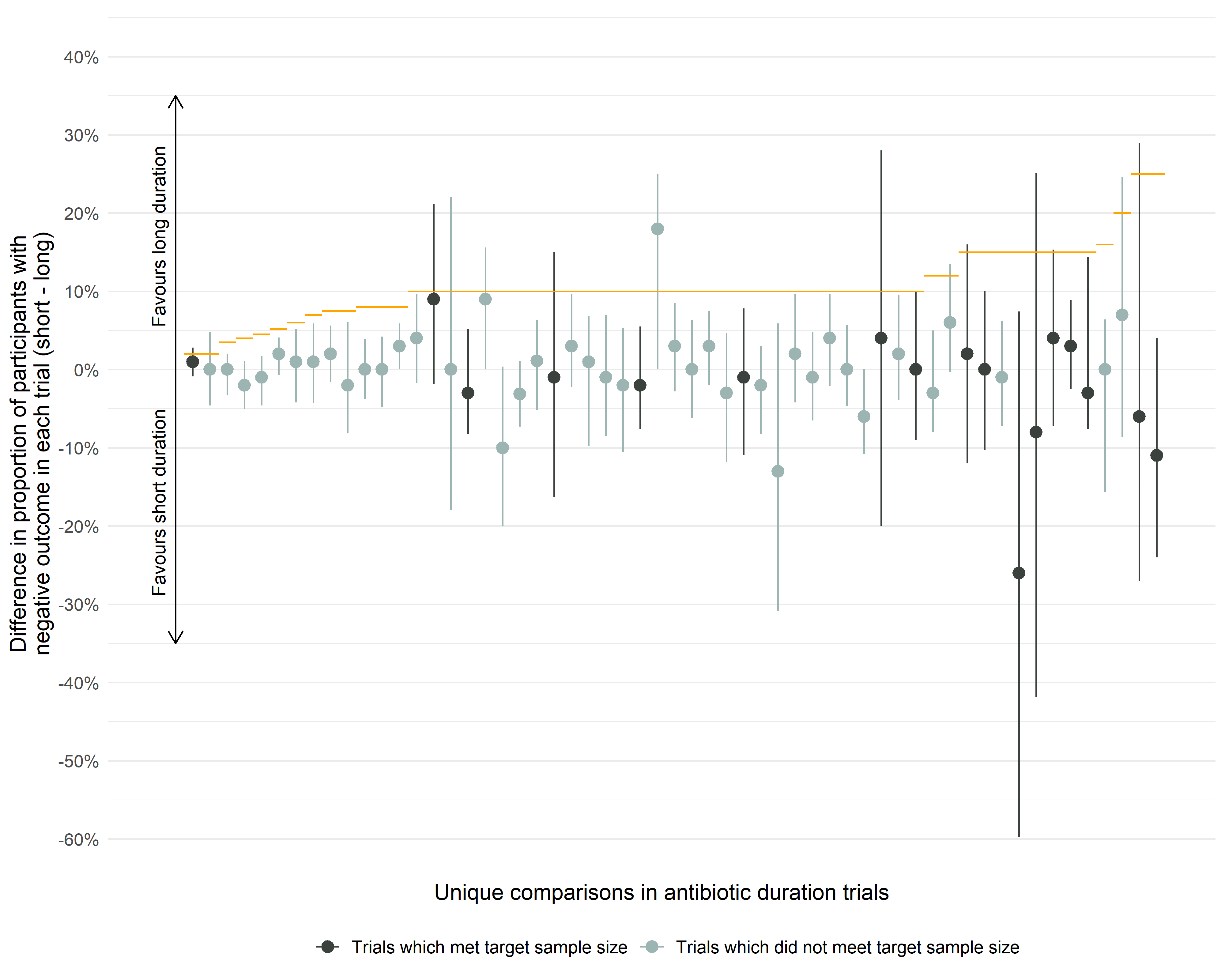


**Supplementary material 5**

**References for summary of antibiotic duration recommendations for bacterial infections from major guidelines (Table 1)**

1. Shulman ST, Bisno AL, Clegg HW, *et al.* Clinical Practice Guideline for the Diagnosis and Management of Group A Streptococcal Pharyngitis: 2012 Update by the Infectious Diseases Society of America. *Clinical Infectious Diseases* 2012; **55**: e86–102.

2. Recommendations | Sore throat (acute): antimicrobial prescribing | Guidance | NICE. 2018. Available at: https://www.nice.org.uk/guidance/ng84/chapter/Recommendations#choice-of-antibiotic. Accessed June 28, 2023.

3. The WHO AWaRe (Access, Watch, Reserve) antibiotic book. Available at: https://www.who.int/publications-detail-redirect/9789240062382. Accessed June 28, 2023.

4. Lieberthal AS, Carroll AE, Chonmaitree T, *et al.* The Diagnosis and Management of Acute Otitis Media. *Pediatrics* 2013; **131**: e964–99.

5. Recommendations | Otitis media (acute): antimicrobial prescribing | Guidance | NICE. 2018. Available at: https://www.nice.org.uk/guidance/ng91/chapter/recommendations#children-and-young-people-who-may-be-less-likely-to-benefit-from-antibiotics-those-not-covered-by. Accessed June 28, 2023.

6. Suzuki HG, Dewez JE, Nijman RG, *et al*. Clinical practice guidelines for acute otitis media in children: a systematic review and appraisal of European national guidelines. *BMJ Open* 2020; **10**: e035343.

7. Chow AW, Benninger MS, Brook I, *et al.* IDSA Clinical Practice Guideline for Acute Bacterial Rhinosinusitis in Children and Adults. *Clinical Infectious Diseases* 2012; **54**: e72–112.

8. Fokkens WJ, Lund VJ, Hopkins C, *et al.* European Position Paper on Rhinosinusitis and Nasal Polyps 2020. *Rhinology* 2020; **58**: 1–464.

9. Bradley JS, Byington CL, Shah SS, *et al.* The Management of Community-Acquired Pneumonia in Infants and Children Older Than 3 Months of Age: Clinical Practice Guidelines by the Pediatric Infectious Diseases Society and the Infectious Diseases Society of America. *Clinical Infectious Diseases* 2011; **53**: e25–76.

10. Recommendations | Pneumonia (community-acquired): antimicrobial prescribing | Guidance | NICE. 2019. Available at: https://www.nice.org.uk/guidance/ng138/chapter/Recommendations. Accessed June 28, 2023.

11. Woodhead M, Blasi F, Ewig S, *et al.* Guidelines for the management of adult lower respiratory tract infections-full version. *Clinical microbiology and infection* 2011; **17**: E1–59.

12. Kalil AC, Metersky ML, Klompas M, *et al.* Management of Adults With Hospital-acquired and Ventilator-associated Pneumonia: 2016 Clinical Practice Guidelines by the Infectious Diseases Society of America and the American Thoracic Society. *Clinical Infectious Diseases* 2016; **63**: e61–111.

13. Torres A, Niederman MS, Chastre J, *et al.* International ERS/ESICM/ESCMID/ALAT guidelines for the management of hospital-acquired pneumonia and ventilator-associated pneumonia: Guidelines for the management of hospital-acquired pneumonia (HAP)/ventilator-associated pneumonia (VAP) of the European Respiratory Society (ERS), European Society of Intensive Care Medicine (ESICM), European Society of Clinical Microbiology and Infectious Diseases (ESCMID) and Asociación Latinoamericana del Tórax (ALAT). *Eur Respir J* 2017; **50**: 1700582.

14. Recommendations | Chronic obstructive pulmonary disease (acute exacerbation): antimicrobial prescribing | Guidance | NICE. 2018. Available at: https://www.nice.org.uk/guidance/ng114/chapter/Recommendations. Accessed June 28, 2023.

15. Wedzicha JA, Miravitlles M, Hurst JR, *et al.* Management of COPD exacerbations: a European Respiratory Society/American Thoracic Society guideline. *European Respiratory Journal* 2017; **49**. Available at: https://erj.ersjournals.com/content/49/3/1600791. Accessed June 28, 2023.

16. Gupta K, Hooton TM, Naber KG, *et al.* International Clinical Practice Guidelines for the Treatment of Acute Uncomplicated Cystitis and Pyelonephritis in Women: A 2010 Update by the Infectious Diseases Society of America and the European Society for Microbiology and Infectious Diseases. *Clinical Infectious Diseases* 2011; **52**: e103–20.

17. Recommendations | Urinary tract infection (lower): antimicrobial prescribing | Guidance | NICE. 2018. Available at: https://www.nice.org.uk/guidance/ng109/chapter/Recommendations. Accessed June 28, 2023.

18. EAU Guidelines on Urological Infections - THE GUIDELINE - Uroweb. *Uroweb - European Association of Urology*. Available at: https://uroweb.org/guidelines/urological-infections/chapter/the-guideline. Accessed June 28, 2023.

19. Hooton TM, Bradley SF, Cardenas DD, *et al.* Diagnosis, Prevention, and Treatment of Catheter-Associated Urinary Tract Infection in Adults: 2009 International Clinical Practice Guidelines from the Infectious Diseases Society of America. *Clinical Infectious Diseases* 2010; **50**: 625–63.

20. Health TNI for, Excellence (NICE) C. Recommendations | Urinary tract infection (catheter-associated): antimicrobial prescribing | Guidance | NICE. Available at: https://www.nice.org.uk/guidance/ng113/chapter/Recommendations. Accessed November 14, 2022.

21. Osmon DR, Berbari EF, Berendt AR, *et al.* Diagnosis and Management of Prosthetic Joint Infection: Clinical Practice Guidelines by the Infectious Diseases Society of Americaa. *Clinical Infectious Diseases* 2013; **56**: e1–25.

22. Liu C, Bayer A, Cosgrove SE, *et al.* Clinical Practice Guidelines by the Infectious Diseases Society of America for the Treatment of Methicillin-Resistant Staphylococcus aureus Infections in Adults and Children: Executive Summary. *Clinical Infectious Diseases* 2011; **52**: 285–92.

23. British National Formulary (BNF) | Musculoskeletal system infections, antimicrobial therapy | Treatment Summaries | NICE [Internet]. 2020. Available at: https://bnf.nice.org.uk/treatment-summaries/musculoskeletal-system-infections-antibacterial-therapy/. Accessed June 28, 2023.

24. Concia E, Prandini N, Massari L, *et al.* Osteomyelitis: clinical update for practical guidelines. *Nuclear Medicine Communications* 2006; **27**: 645.

25. Ravn C, Neyt J, Benito N, *et al.* Guideline for management of septic arthritis in native joints (SANJO). *J Bone Jt Infect* 2023; **8**: 29–37.

26. Mermel LA, Allon M, Bouza E, *et al.* Clinical Practice Guidelines for the Diagnosis and Management of Intravascular Catheter-Related Infection: 2009 Update by the Infectious Diseases Society of America. *Clinical Infectious Diseases* 2009; **49**: 1–45.

27. Recommendations | Neonatal infection: antibiotics for prevention and treatment | Guidance | NICE. 2021. Available at: https://www.nice.org.uk/guidance/ng195/chapter/Recommendations#duration-of-antibiotic-treatment-for-early-onset-neonatal-infection. Accessed June 28, 2023.

28. Kenna MA. Acute Otitis Media — The Long and the Short of It. *https://doi-org.libproxy1.nus.edu.sg/101056/NEJMe1614712* 2016. Available at: http://www.nejm.org/doi/full/10.1056/NEJMe1614712. Accessed November 9, 2022.

29. Tunkel AR, Hartman BJ, Kaplan SL, *et al.* Practice Guidelines for the Management of Bacterial Meningitis. *Clinical Infectious Diseases* 2004; **39**: 1267–84.

30. Recommendations | Meningitis (bacterial) and meningococcal septicaemia in under 16s: recognition, diagnosis and management | Guidance | NICE. 2010. Available at: https://www.nice.org.uk/guidance/cg102/chapter/Recommendations. Accessed June 28, 2023.

31. Beek D van de, Cabellos C, Dzupova O, *et al.* ESCMID guideline: diagnosis and treatment of acute bacterial meningitis. *Clinical Microbiology and Infection* 2016; **22**: S37–62.

32. Stevens DL, Bisno AL, Chambers HF, *et al.* Practice Guidelines for the Diagnosis and Management of Skin and Soft Tissue Infections: 2014 Update by the Infectious Diseases Society of America. *Clinical Infectious Diseases* 2014; **59**: e10–52.

33. Recommendations | Cellulitis and erysipelas: antimicrobial prescribing | Guidance | NICE. 2019. Available at: https://www.nice.org.uk/guidance/ng141/chapter/Recommendations#choice-of-antibiotic. Accessed June 28, 2023.

34. Solomkin JS, Mazuski JE, Bradley JS, *et al.* Diagnosis and Management of Complicated Intra-Abdominal Infection in Adults and Children: Guidelines by the Surgical Infection Society and the Infectious Diseases Society of America. *Surgical Infections* 2010; **11**: 79–109.

35. Montravers P, Dupont H, Leone M, *et al.* Guidelines for management of intra-abdominal infections. *Anaesthesia Critical Care & Pain Medicine* 2015; **34**: 117–30.

36. Sartelli M, Coccolini F, Kluger Y, *et al.* WSES/GAIS/SIS-E/WSIS/AAST global clinical pathways for patients with intra-abdominal infections. *World Journal of Emergency Surgery* 2021; **16**: 49.

37. Mazuski JE, Tessier JM, May AK, *et al.* The Surgical Infection Society Revised Guidelines on the Management of Intra-Abdominal Infection. *Surgical Infections* 2017; **18**: 1–76.

38. Bratzler DW, Dellinger EP, Olsen KM, *et al.* Clinical Practice Guidelines for Antimicrobial Prophylaxis in Surgery. *Surgical Infections* 2013; **14**: 73–156.

39. Recommendations | Surgical site infections: prevention and treatment | Guidance | NICE. 2019. Available at: https://www.nice.org.uk/guidance/ng125/chapter/Recommendations. Accessed June 28, 2023.

40. ECDC., Public Health England., Institut de Veillle Sanitaire. *Systematic review and evidence-based guidance on perioperative antibiotic prophylaxis.* LU: Publications Office; 2013. Available at: https://data.europa.eu/doi/10.2900/85936. Accessed June 28, 2023.
