# Supplementary figures and images for: Antibiotic duration for common bacterial infections—a systematic review
Source: JAC Antimicrob Resist. 2025 Jan 29;7(1):dlae215. doi: 10.1093/jacamr/dlae215 (PMC11775593; doi:10.1093/jacamr/dlae215)

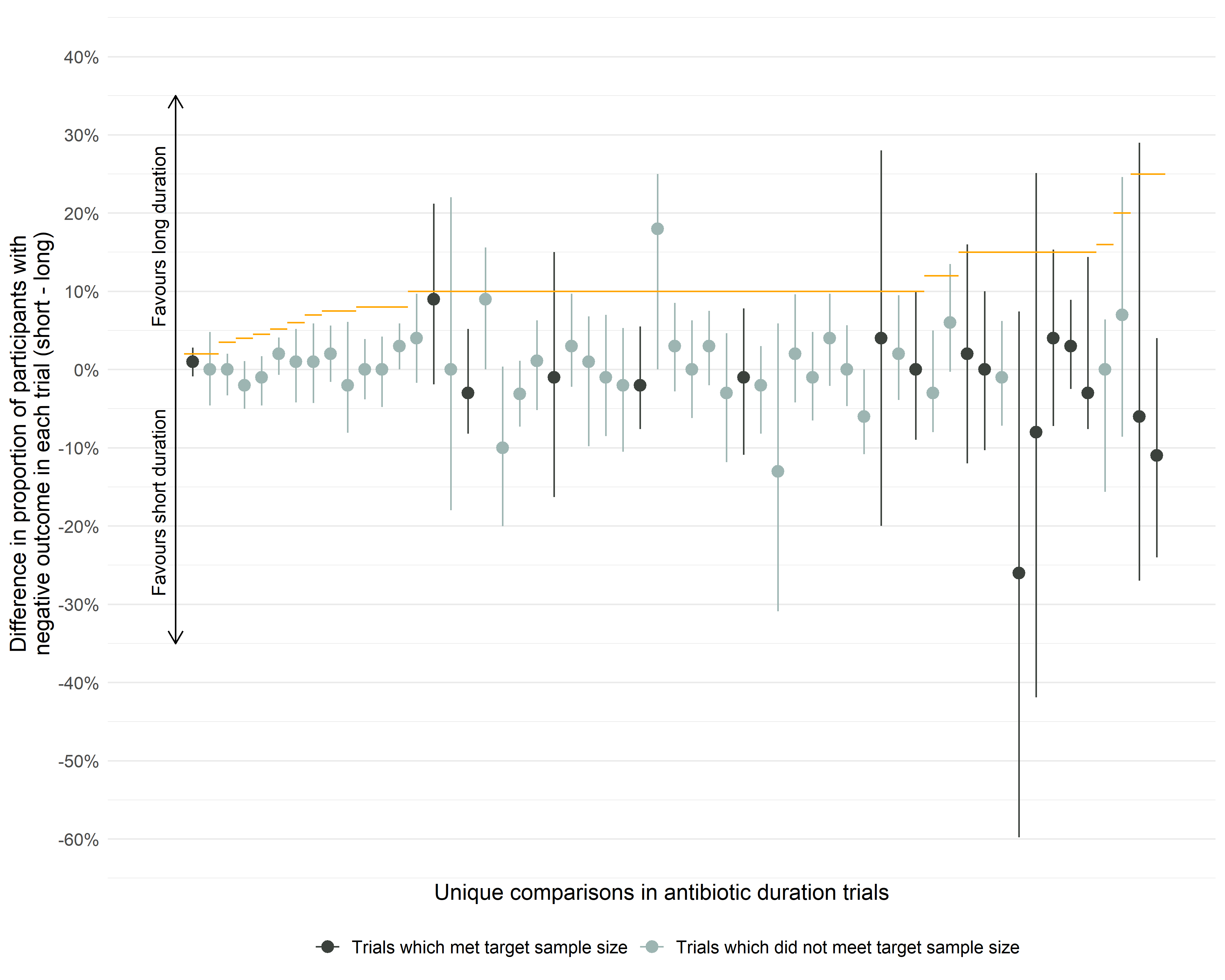

Supplement: dlae215_Supplementary_Data [file dlae215_supplementary_data.zip › fig_suppl_effect_ni.png]
